# T-Cell Engagers Targeting *BCMA*, *GPRC5D*, or *FcRH5* in Relapsed/Refractory Multiple Myeloma: The Landscape Beyond CAR-T Cell Therapy-A Systematic Review and Network Meta-Analysis

**DOI:** 10.3390/ijms27167179

**Published:** 2026-08-11

**Authors:** Shreya Shambhavi, Aksa Alina Joy, Harmanjeet Singh, Terence Amonica, Astha Grover, Tanya Singh, Sharon Susan Paul, Tiffany Pompa

**Affiliations:** 1 Department of Internal Medicine, Rutgers Health-RWJ Barnabas Health, Community Medical Center, 99 NJ-37, Toms River, NJ 08755, USA; aksaalinajoy@gmail.com (A.A.J.); sharon.paul2@rwjbh.org (S.S.P.); 2 Mahatma Gandhi Memorial Medical College, Jamshedpur 831020, India; harman.329@gmail.com (H.S.); tanyasingh6996@gmail.com (T.S.); 3 Rowan-Virtua School of Osteopathic Medicine, Stratford, NJ 08084, USA; amonic34@rowan.edu; 4 School of Medical Sciences and Research, Greater Noida 201306, India; groverastha@gmail.com; 5 Department of Hematology and Oncology, Rutgers Health-RWJ Barnabas Health, Community Medical Center, 99 NJ-37, Toms River, NJ 08755, USA; tpompa@gmail.com

**Keywords:** *BCMA*, *GPRC5D*, *FcRH5*, relapsed/refractory multiple myeloma

## Abstract

Relapsed/refractory multiple myeloma is marked by frequent triple-class refractoriness and poor survival, whereas bispecific antibodies targeting *BCMA*, *GPRC5D*, or *FcRH5* show meaningful activity even in heavily pretreated and post-BCMA disease. This meta-analysis evaluates and compares the efficacy and safety of these agents in RRMM using direct and indirect evidence. We manually searched seven databases and identified 44 studies for quantitative analysis. Forest plots were created using R 4.1.1 software. Compared to Standard of care (SOC), the odds of Objective response rate (ORR) were higher with Talquetamab, Teclistamab, Elranatamab, and Linvoseltamab (5.73, 4.86, 3.84, and 2.63). PFS improved with Teclistamab (HR 0.50, 95% CI 0.36–0.55), Talquetamab (0.50, 0.36–0.55), Elranatamab (0.45, 0.36–0.55), and Linvoseltamab (0.23, 0.17–0.31). Linvoseltamab and Elranatamab showed numerically longer OS relative to SOC (HR 0.41, 0.24–0.70 and HR 0.58, 0.43–0.78, respectively), but numerically shorter OS with Teclistamab (HR 1.82, 1.37–2.42) and Talquetamab (HR 1.75, 1.20–2.57). In pooled single-arm data, Talquetamab had the highest ORR (72%) and CRS rate (68%); Teclistamab showed an ORR of 61% with a CRS rate of 61% and Linvoseltamab showed an ORR of 60% with a CRS rate of 51%. Cevostamab and Elranatamab had ORRs of 49% and 56% with CRS rates of 61% and 52%, respectively. In RRMM, all bispecific antibodies showed superior ORR along with improved PFS compared to SOC. Linvoseltamab and Elranatamab showed numerically longer OS relative to SOC, whereas Talquetamab and Teclistamab showed numerically shorter OS versus SOC, within the constraints of adjusted cross-trial comparisons.

## 1. Introduction

Relapsed/refractory multiple myeloma (RRMM) has been defined as an advanced disease that either progresses during treatment within 60 days of the last regimen administered or recurs after an initial response to therapy [1,2]. Although standard treatments for multiple myeloma patients include proteasome inhibitors (PIs), immunomodulatory drugs (IMiDs), and anti-*CD38* monoclonal antibodies, a growing subset of patients develops triple-class refractory (TCR) disease (refractory to ≥1 PI, ≥1 IMiD, and an anti-*CD38* antibody), which indicates poor outcomes with overall survival frequently less than a year in observational cohorts [3]. The need for more effective immune-based treatments has been highlighted by recent prospective data among triple-class-exposed (TCE) RRMM patients, which revealed limited outcomes (ORR ~32%, median OS 13.8 months) [4].

Idecabtagene vicleucel (Ide-cel) and Ciltacabtagene autoleucel (Cilta-cel), two BCMA-directed Chimeric T-cell therapy (CAR-T) therapies, have transformed the clinical and investigational landscape through the production of deep responses in heavily pretreated RRMM. Ide-cel also demonstrated durable activity in KarMMa with persistent efficacy in long-term follow-up data. In extended CARTITUDE-1 follow-up, Cilta-cel showed longer survival (median OS 60.7 months at ~5-year follow-up in treated patients) [5,6]. There is currently no single definitive post-CAR-T standard treatment, and progression following *BCMA* CAR-T therapy is still common. Retreatment or sequencing strategies that include T-cell-redirecting agents, switching targets, and *BCMA*-directed strategies are backed by emerging data. Resistance may involve antigen escape and other tumor-intrinsic adaptive mechanisms, yet response times after salvage may be shorter [7,8].

Importantly, the equity implications of these novel therapies are increasingly recognized. African American, Hispanic, and Asian patients are underrepresented in pivotal cellular therapy trials, and recent studies suggest disparities in access and potential differences in inflammatory toxicity and outcomes. Some multicenter datasets report comparable efficacy when access is achieved, emphasizing that structural barriers may be a key driver of outcome gaps [9,10,11]. This further emphasizes the need for inclusion of real-world data representing diverse ethnic and socioeconomic groups in conjunction with data from clinical trials while trying to gauge the efficacy of bispecific antibodies (BsAbs) in the treatment of RRMM.

Targeting myeloma antigens such as *BCMA* (Teclistamab; Elranatamab), *GPRC5D* (Talquetamab), and *FcRH5* (Cevostamab), bispecific antibodies (BsAbs) or T-cell redirecting therapies have rapidly grown into a novel option for RRMM. Clinical trials have shown substantial activity even in post-*BCMA* exposed settings [12,13,14,15]. However, because of diverse populations, prior exposure to *BCMA*, and developing sequencing techniques (including BsAbs as bridging to CAR-T), comparative efficacy and survival standards across individual BsAbs remain ambiguous [13,14,15,16]. Therefore, the objective of this meta-analysis is to draw robust and thorough comparisons between the different bispecific antibodies in terms of efficacy, their impact on survival outcomes in RRMM, and the differences in their adverse effect profiles by including data from pivotal trials and real-world databases.

## 2. Methods

This systematic review and meta-analysis was conducted and reported in accordance with the recommendations and criteria outlined in the Preferred Reporting Items for Systematic Reviews and Meta-Analyses (PRISMA) guidelines [17]. After formulating the clinical question and refining it into a focused systematic review question using the PICOS framework (Patients, Interventions, Comparators, Outcomes, and Study design), we conducted a search of major medical databases, including PubMed, Embase, Cochrane Library, Scopus, Google Scholar, and Web of Science in English from inception through January 2026. The search was conducted using the following PICOS format. P: adult participants (age > 18 years) who had relapsed/refractory multiple myeloma (triple-refractory to anti-CD38 therapy, immunomodulator, and a proteasome inhibitor, or penta-refractory), I: Intervention group treated with bispecific antibodies targeting BCMA × CD3 or GPRC5D × CD3 or FcRH5 × CD3 receptors; C: control group treated with Standard of Care (SOC); O: objective response rate (ORR), ≥very good partial response (VGPR), infections, cytokine release syndrome (CRS), progression-free survival (PFS), overall survival (OS), immune effector cell-associated neurotoxicity syndrome (ICANS), ≥grade 3 neutropenia, ≥grade 3 thrombocytopenia and ≥grade 3 anemia; S: single-arm clinical trials, retrospective studies, matching-adjusted indirect comparison (MAIC) studies, inverse probability of treatment weighting (IPTW) comparison studies. Our meta-analysis was prospectively registered in PROSPERO (ID: CRD420261347139).

### 2.1. Eligibility Criteria

We conducted a systematic review of relevant studies published in these databases from inception to January 2026, available in English, and screened clinical trials, cohort studies and comparative studies. Studies were included if: (1) they involved humans and ≥18-year-old participants were enrolled; (2) they enrolled heavily pretreated RRMM populations with a high proportion of triple/penta-refractory patients who were treated with bispecific antibodies targeting *BCMA* × *CD3* or *GPRC5D* × *CD3* or *FcRH5* × *CD3* receptors; and (3) ≥1 of the following parameters are available or can be calculated: ORR, ≥VGPR, ≥CR, infections, CRS, PFS, OS, ICANS, ≥grade 3 neutropenia, ≥grade 3 thrombocytopenia, and ≥grade 3 anemia. Studies were excluded if: (1) they were non-human studies (cell culture, animal models); (2) they were case reports, protocol articles, reviews, news articles, letters to the editor, editorials; (3) they were duplicates; (4) they were written in a language other than English and had no English translations; (5) the data on clinical outcome measures (odds ratios, 95% CIs) were incomplete or the outcome measures could not be calculated with the available data.

### 2.2. Outcome Measures

The primary outcomes of this systematic review and meta-analysis were ORR, ≥VGPR, PFS, and OS. Secondary outcomes were ≥CR, infections, CRS, ICANS, ≥grade 3 neutropenia, ≥grade 3 thrombocytopenia, and ≥grade 3 anemia in patients with RRMM who were treated with bispecific antibodies.

### 2.3. Data Synthesis and Analysis

The selected studies were reviewed, and the data were independently extracted by two researchers (SS and HS). Discrepancies between them were resolved by consensus among all authors.

We extracted study characteristics (authors, year, region, design, sample size, age, outcomes) and entered them into an Excel spreadsheet (version 2021; Microsoft Corporation, Redmond, WA, USA). We structured the narrative synthesis by patient and exposure characteristics and summarized effect sizes for each outcome. When ORs and 95% CIs were not reported, we calculated crude values from 2 × 2 tables. We considered differences statistically significant at *p* < 0.05.

To assess heterogeneity in effect measures, we used the I^2^ statistic (per Cochrane Handbook guidelines), where I^2^ reflects the percentage of total variation attributable to between-study heterogeneity: <30% (low), 30–60% (moderate), 60–75% (substantial), and >75% (considerable) [18]. We explored sources of heterogeneity and performed sensitivity analyses via the leave-one-out method. Given expected variability, we used the random-effects model of DerSimonian and Laird to pool ORs and 95% CIs [19]. A subgroup analysis on the basis of prior BCMA exposure was conducted. All analyses were performed in R (version 4.4.1). Since this study was a meta-analysis of published studies, institutional review board approval was not required.

### 2.4. Study Quality Assessment

The quality of observational studies was evaluated using the Newcastle-Ottawa scale (NOS), and that of single-arm clinical trials and comparative studies using the ROBINS-I tool [20,21]. Studies scoring 7–9 were classified as high quality, 4–6 as moderate quality, and ≤3 as low quality using the NOS. The ROBINS-I tool classified studies as having low, moderate, serious, or critical risk of bias across seven domains (confounding, selection of participants, classification of interventions, deviations from intended interventions, missing data, measurement of outcomes, and selection of the reported result). Two reviewers (SS and HS) independently assessed the risk of bias, with any discrepancies resolved through consensus among all authors.

Table 1 summarizes the characteristics of the included single-arm trials and retrospective studies. Table 2 summarizes the characteristics of the comparative studies.

### 2.5. Search Results

**Figure 1:**
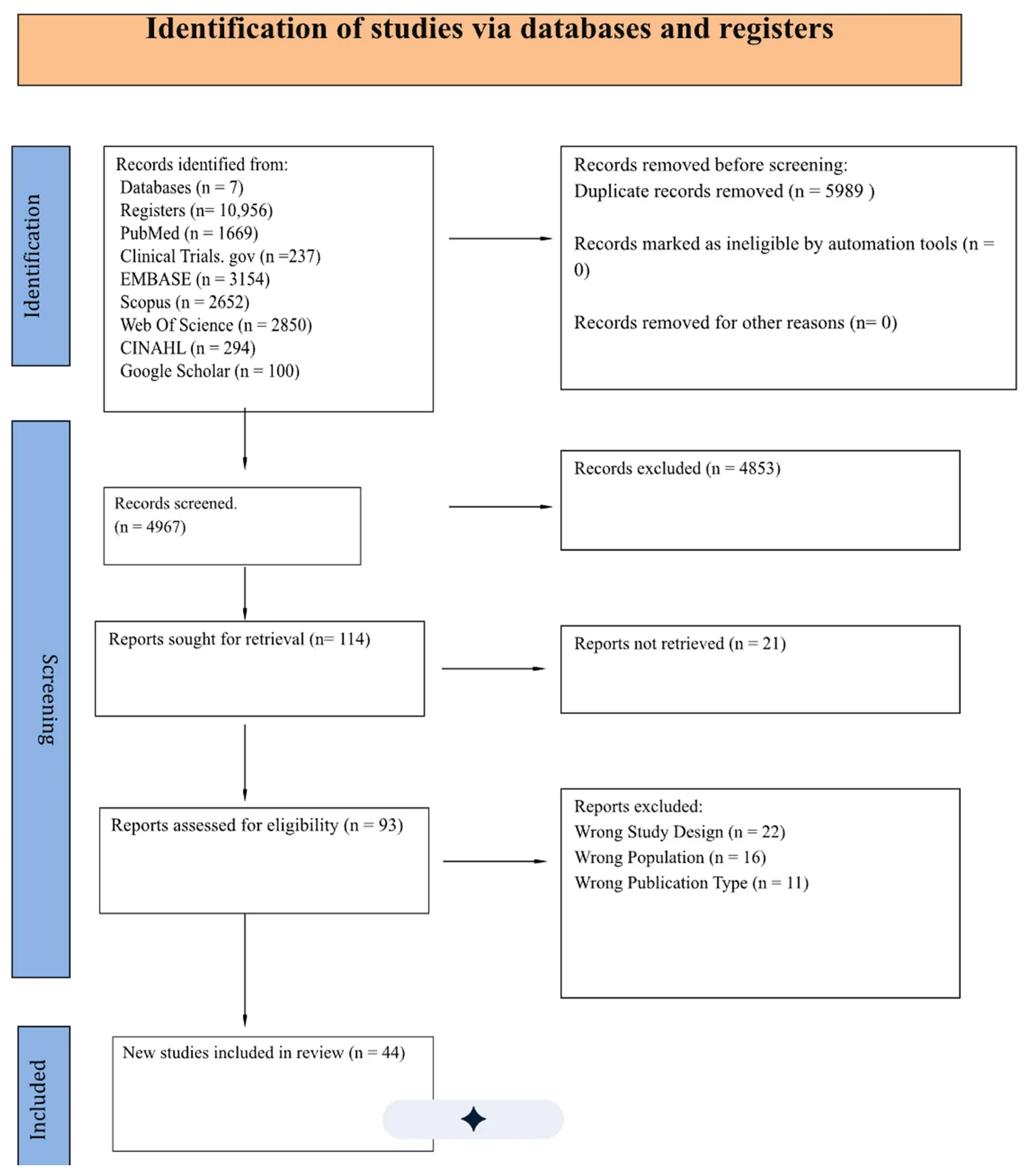
PRISMA flowchart.

A total of 10,956 articles were collected from seven databases. Before the screening of the potential articles, 5989 duplicate articles were removed, and a total of 4967 records were screened; 4853 were excluded at the title/abstract screening stage. Of the remaining reports, 114 were sought for retrieval, of which 21 reports could not be retrieved. A total of 93 articles were assessed for eligibility in the final review process, of which 22 were excluded for the wrong study design, 16 for the wrong population, and 11 for the wrong publication type. The remaining 44 articles (19 single-arm trials with 21 cohorts, 15 retrospective studies, and 10 comparative studies) were included in this systematic review and quantitative meta-analysis. Two studies each contributed two independent cohorts, yielding a total of 46 cohorts for the final analysis. The systematic approach adopted for the study selection is illustrated in Figure 1 of the PRISMA flow diagram. The detailed search strategy with corresponding results is summarized in Table S1 in the Supplementary Table File. Figures S1 and S2A,B in the Supplementary File depict the overall risk of bias of the included studies.

## 3. Results

### 3.1. Network Meta-Analysis

A total of 10 comparative cohorts (8 MAIC and 2 IPTW) comparing different bispecifics with SOC were included in the indirect (network) meta-analysis [55,56,57,58,59,60,61,62].

### 3.2. Objective Response Rate (ORR) and ≥Very Good Partial Response (≥VGPR)

**Figure 2:**
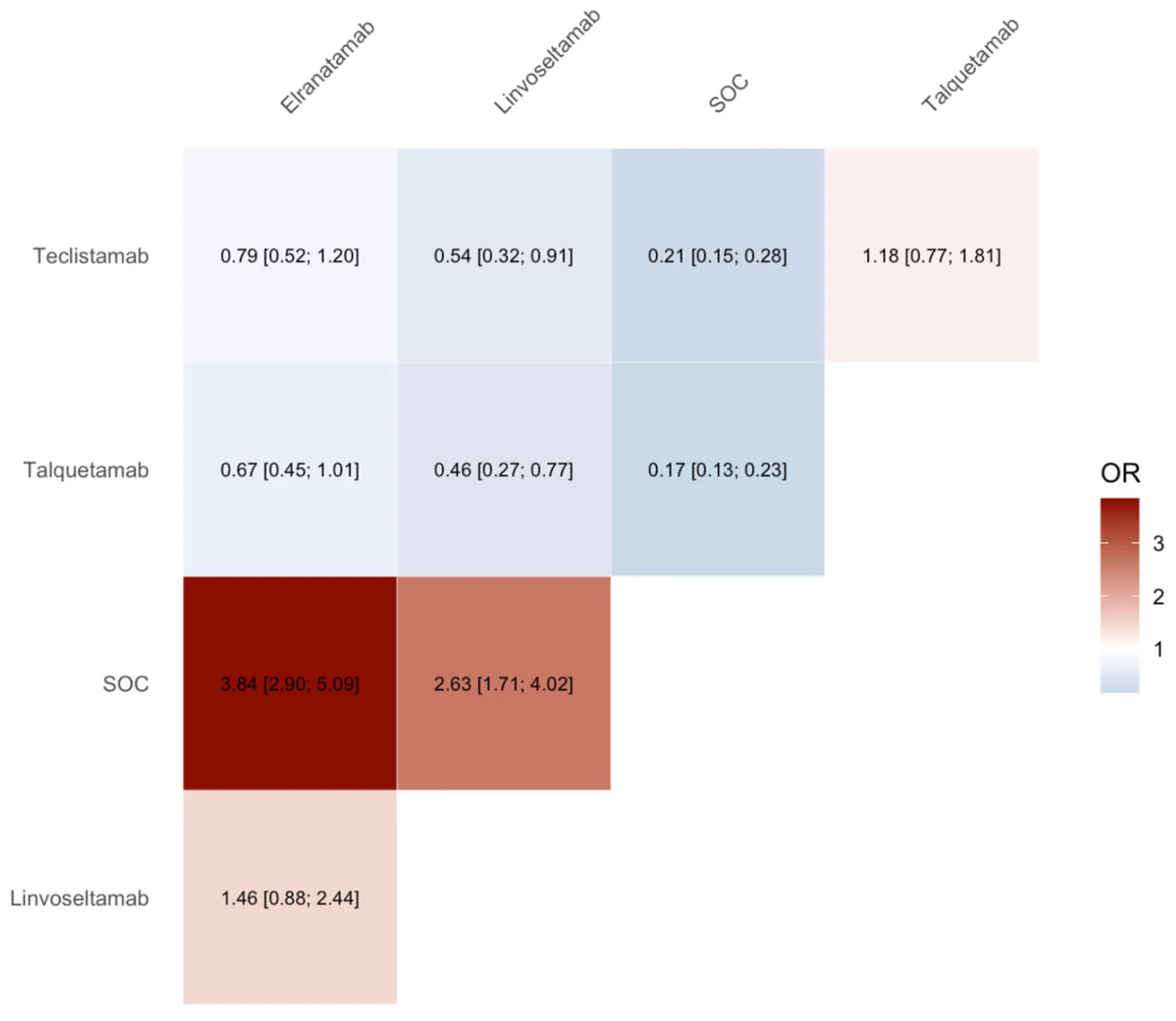
Network heat map of pairwise odds ratio for ORR comparing bispecific agents and Standard of Care regimens.

As depicted in the network heat map in Figure 2, after inverting the reference direction, the odds of achieving an ORR were 4.86-fold higher with Teclistamab (95% CI: 3.58–6.59), 5.73-fold higher with Talquetamab (95% CI: 4.26–7.71), 3.84-fold higher with Elranatamab (95% CI: 2.90–5.09), and 2.63-fold higher with Linvoseltamab (95% CI: 1.71–4.02), compared with SOC. Also, when Talquetamab was compared with other bispecifics (BsAbs), it showed significantly higher ORR than Linvoseltamab (OR 2.17, 95% CI 1.30–3.70), a numerically higher ORR than both Teclistamab (OR 1.18, 95% CI: 0.77–1.81) and Elranatamab (OR 1.49, 95% CI: 0.92–2.21), although neither comparisons reached statistical significance. Teclistamab showed significantly higher ORR than Linvoseltamab (OR 1.85, 95% CI: 1.10–3.13) but comparable ORR to Elranatamab (OR 1.27, 95% CI: 0.83–1.92).

Subgroup analysis was performed based on prior *BCMA* exposure, as shown in Supplementary File Figure S3. In the *BCMA*-naive cohort, Linvoseltamab, Talquetamab, and Teclistamab achieved significantly higher response rates than SOC. Among the bispecifics, Teclistamab had a statistically significantly higher ORR than Linvoseltamab; Talquetamab showed a numerically higher ORR than Linvoseltamab with borderline significance. Teclistamab and Talquetamab (and Elranatamab vs. either) had broadly similar ORRs. The *BCMA*-exposed cohort had only two studies, limiting the feasibility of indirect analysis in this cohort.

The network heat map in Figure S4 showed that the odds of achieving ≥VGPR were 8.62-fold higher with Elranatamab, 5.38-fold higher with Linvoseltamab, 12.50-fold higher with Teclistamab, and 10.00-fold higher with Talquetamab compared with SOC. Detailed subgroup analysis is mentioned in Figure S5.

### 3.3. Progression-Free Survival (PFS)

**Figure 3:**
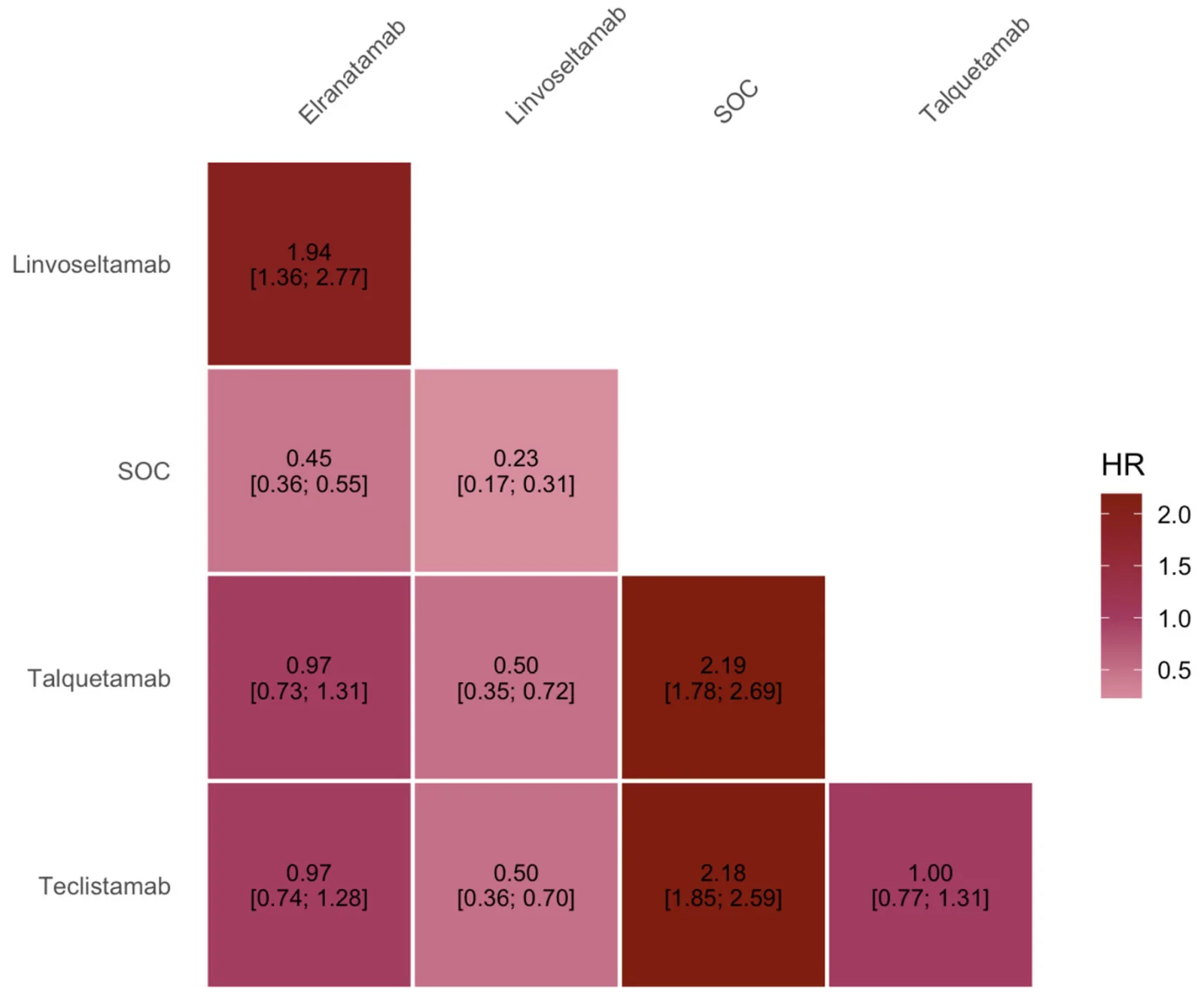
Network heat map of pairwise hazard ratio for comparing PFS among bispecific agents and SOC regimens.

The network heat map for PFS in Figure 3 shows that all bispecific antibodies were associated with longer PFS than SOC, with the highest benefit observed for Linvoseltamab (HR 0.23, 95% CI: 0.17–0.31 vs. SOC), followed by Elranatamab (HR 0.45, 95% CI: 0.36–0.55), Teclistamab (HR 0.46, 95% CI: 0.39–0.54), and Talquetamab (HR 0.46, 95% CI: 0.37–0.56). When compared amongst each other, Linvoseltamab showed a significantly lower risk of progression or death than Talquetamab (HR 0.50, 95% CI: 0.35–0.72) and Teclistamab (HR 0.50, 95% CI: 0.36–0.70). Linvoseltamab showed shorter PFS relative to Elranatamab (HR 1.94, 95% CI: 1.36–2.77). There was no difference observed in PFS either between Talquetamab and Teclistamab (HR 1.00, 95% CI: 0.77–1.31), or between Talquetamab and Elranatamab (HR 0.97, 95% CI: 0.73–1.31), and between Teclistamab and Elranatamab (HR 0.97, 95% CI: 0.74–1.28).

Upon subgroup analysis with prior *BCMA* exposure, as shown in Figures S6 and S7 in the Supplementary File, all bispecifics improved PFS in the *BCMA*-naive group compared with SOC, with Linvoseltamab having the greatest effect (HR 0.23, 95% CI: 0.17–0.31), followed by Elranatamab (HR 0.42, 95% CI: 0.33–0.54), Talquetamab (HR 0.47, 95% CI: 0.33–0.67), and Teclistamab (HR 0.46, 95% CI: 0.38–0.55). In head-to-head comparisons, Linvoseltamab was superior to Elranatamab (HR 0.54, 95% CI: 0.37–0.81), Talquetamab (HR 0.49, 95% CI: 0.31–0.78) and Teclistamab (HR 0.50, 95% CI: 0.35–0.72). Elranatamab, Talquetamab and Teclistamab had broadly similar PFS. In patients previously exposed to *BCMA*-directed agents, Elranatamab showed a longer PFS than SOC (HR 0.52, 95% CI: 0.34–0.79), and Talquetamab showed a significantly longer PFS than SOC (HR 0.45, 95% CI: 0.35–0.58). The PFS with Talquetamab and Elranatamab did not differ significantly from each other (HR 1.16, 95% CI: 0.71–1.88).

### 3.4. Overall Survival (OS)

**Figure 4:**
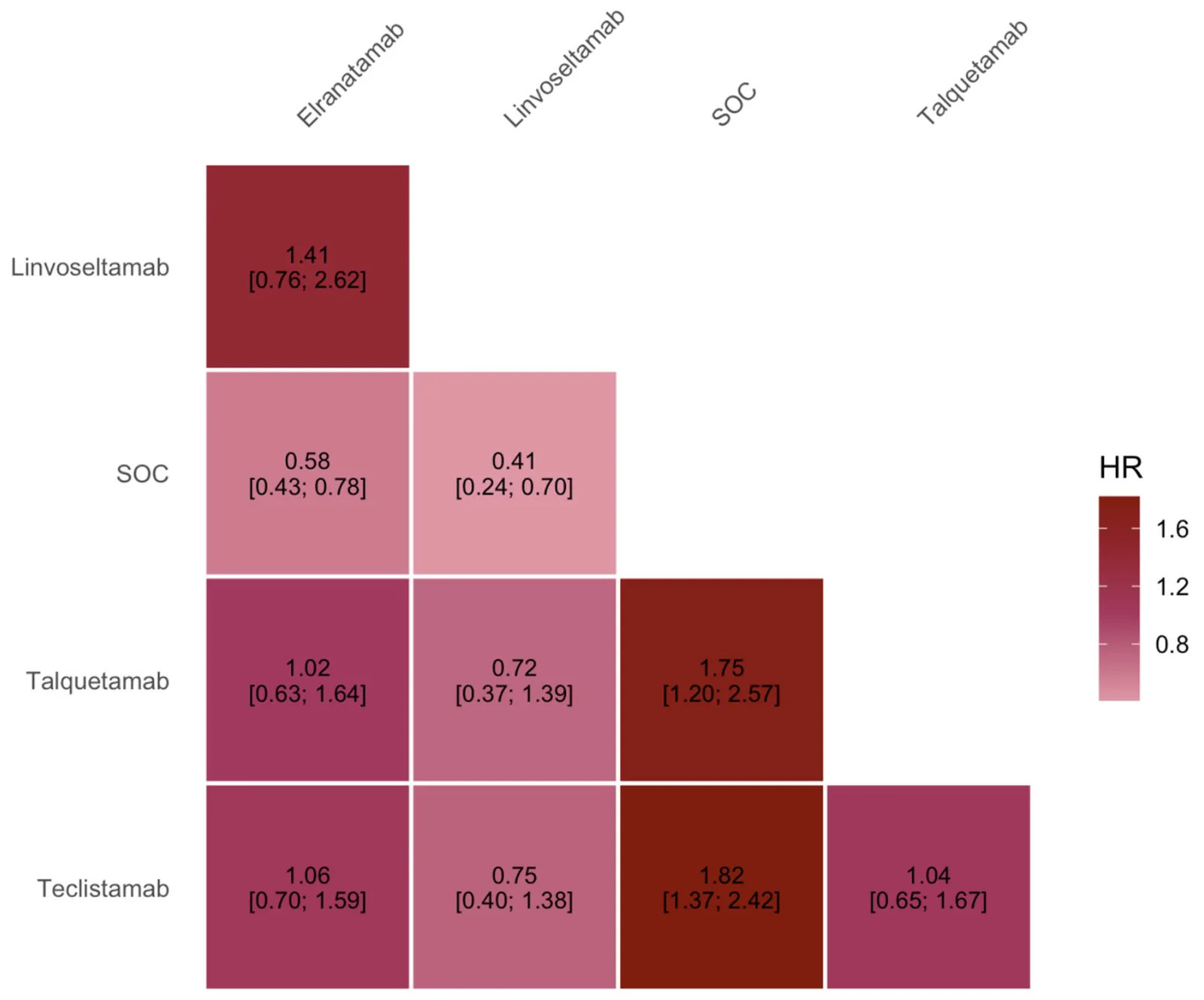
Network heat map of pairwise hazard ratio for comparing OS among bispecific agents and Standard of Care regimens.

The network heat map for OS in Figure 4 shows that both Linvoseltamab and Elranatamab were associated with longer OS than SOC, with the lowest HR observed for Linvoseltamab (HR 0.41, 95% CI: 0.24–0.70), followed by Teclistamab (HR 0.55, 95% CI: 0.41–0.73), Talquetamab (HR 0.57, 95% CI: 0.39–0.83), and Elranatamab (HR 0.58, 95% CI: 0.43–0.78). In head-to-head comparisons, OS did not differ significantly among the four bispecific agents. Talquetamab showed a similar OS when compared with Linvoseltamab (HR 0.72, 95% CI: 0.37–1.39) and Elranatamab (HR 1.02, 95% CI: 0.63–1.64). Also, Teclistamab demonstrated a comparable OS to Linvoseltamab (HR 0.75, 95% CI: 0.40–1.38) and Talquetamab (HR 1.04, 95% CI: 0.65–1.67). Elranatamab and Teclistamab also showed similar OS (HR 1.06, 95% CI: 0.70–1.59), while Elranatamab showed a numerically higher hazard of death compared with Linvoseltamab (HR 1.41, 95% CI: 0.76–2.62), although not statistically significant.

Upon subgroup analysis with prior *BCMA* exposure, as shown in the Supplementary Files S8 and S9, in the *BCMA*-naive cohort, Linvoseltamab (HR 0.41, 95% CI 0.23–0.72), Elranatamab (HR 0.54, 95% CI 0.38–0.76), and Teclistamab (HR 0.55, 95% CI 0.41–0.74) were each associated with significantly longer OS than SOC. Talquetamab showed a numerically longer OS than SOC but it did not reach statistical significance (HR 0.69, 95% CI: 0.39–1.22). Among the bispecifics themselves, no pair showed a statistically significant OS advantage. In the *BCMA*-exposed cohort, Talquetamab showed a significantly longer OS as compared to SOC (HR 0.48, 95% CI 0.33–0.70), while Elranatamab showed a numerically longer OS that did not reach statistical significance (HR 0.75, 95% CI 0.45–1.24). OS did not differ significantly between Talquetamab and Elranatamab (HR 1.56, 95% CI 0.83–2.93). This subgroup was limited to three nodes, with no data for Linvoseltamab or Teclistamab, so these indirect estimates could not be interpreted.

### 3.5. Single-Arm Pooled Analysis

A total of 36 studies (21 single-arm trials, 15 retrospective studies) were included in the pooled single-arm analysis.

### 3.6. Talquetamab

Across 13 studies comprising 1494 patients treated with Talquetamab-based regimens, the pooled objective response rate was 72% (95% CI: 68–75%) with moderate between-study heterogeneity (I^2^ = 40%).

≥VGPR was achieved in 37% of 1269 patients (95% CI: 21–57%; I^2^ = 93%) and ≥CR in 18% of 760 patients (95% CI: 11–28%; I^2^ = 85%). The pooled incidence of ICANS was 25% (95% CI: 19–33%) in 577 patients, and CRS was seen in 68% (95% CI: 61–75%) in 1355 patients. The pooled incidences of ≥grade 3 thrombocytopenia, anemia, and neutropenia were 25%, 31%, and 30% among 577 patients, and infections occurred in 58% of 1268 patients (95% CI: 44–71%). Detailed forest plots are summarized in Figures S10–S18.

### 3.7. Teclistamab

Across 11 cohorts comprising 1249 patients treated with Teclistamab-based regimens, the pooled objective response rate was 61% (95% CI: 57–66%) with moderate between-study heterogeneity (I^2^ = 52.3%). ≥VGPR was achieved in 47% of 946 patients (95% CI: 32–62%; I^2^ = 84.0%) and ≥CR in 35% of 836 patients (95% CI: 24–47%; I^2^ = 82.5%). The pooled incidence of ICANS was 7% (95% CI: 5–11%) in 946 patients, CRS occurred in 61% (95% CI: 54–67%) in 946 patients, and ≥grade 3–4 thrombocytopenia, anemia, and neutropenia occurred in 19%, 24%, and 50% among 783, 581, and 823 patients, respectively. Infections occurred in 66% of 946 patients. Detailed forest plots are summarized in Figures S19–S27.

### 3.8. Elranatamab

Across six studies comprising 552 patients treated with Elranatamab-based regimens, the pooled ORR was 56% (95% CI: 44–68%) with moderate between-study heterogeneity (I^2^ = 74.6%).

≥VGPR was achieved in 52% of 303 patients (95% CI: 38–66%; I^2^ = 28.7%) and ≥CR in 32% of 366 patients (95% CI: 19–49%; I^2^ = 61.7%). The pooled incidence of ICANS was 11% (95% CI: 1–58%) in 303 patients; CRS occurred in 74% of 552 patients; and the pooled incidences of ≥grade 3–4 thrombocytopenia, anemia, and neutropenia were 28%, 49%, and 56% among 248, 303, and 303 patients, respectively. Infections occurred in 60% of 224 patients. Detailed forest plots are summarized in Figures S28–S36.

### 3.9. Linvoseltamab

Across two studies comprising 221 patients treated with Linvoseltamab-based regimens, the pooled ORR was 60% (95% CI: 0–100%) with high between-study heterogeneity (I^2^ = 91.5%), and CRS occurred in 51% of 221 patients. ≥VGPR was achieved in 51% of 221 patients (95% CI: 0–100%; I^2^ = 91.9%) and ≥CR in 34% of 221 patients (95% CI: 0–100%; I^2^ = 94.6%) and the pooled incidences of ≥grade 3–4 thrombocytopenia, anemia, and neutropenia were 19%, 30%, 40% among 221, 221, and 221 patients, respectively. Infections occurred in 74% of 221 patients. Detailed forest plots are summarized in Figures S37–S44.

### 3.10. Cevostamab

Across seven studies comprising 458 patients treated with Cevostamab-based regimens, the pooled ORR was 49% (95% CI: 24–74%) with moderate between-study heterogeneity (I^2^ = 86.6%).

≥VGPR was achieved in 27% of 404 patients (95% CI: 20–35%; I^2^ = 34.4%) and ≥CR in 10% of 383 patients (95% CI: 4–24%; I^2^ = 64.4%). The pooled incidence of ICANS was 20% (95% CI: 10–35%) in 331 patients, CRS occurred in 74% of 458 patients, and the pooled incidences of ≥grade 3–4 thrombocytopenia, anemia, and neutropenia were 38%, 29%, 31% among 42, 261, and 261 patients respectively. Infections occurred in 52% of 215 patients. Detailed forest plots are summarized in Figures S45–S53.

### 3.11. Subgroup Analysis of Single-Arm Trials/Studies

In the *BCMA*-naive cohort, ORR was 72% with Talquetamab, 82% with Teclistamab, 62% with Elranatamab, and 39% with Cevostamab; CRS occurred in 66% with Teclistamab, 61% with Elranatamab and 75% with Cevostamab. All the recipients of Talquetamab had prior *BCMA* exposure. In the *BCMA*-exposed cohort, ORR was 72% with Talquetamab, 60% with Teclistamab, and 50% with Elranatamab; CRS occurred in 64%, 57%, and 42% respectively. Detailed forest plots are mentioned in S54–S73.

## 4. Discussion

This meta-analysis explores the efficacy of different bispecific antibodies in RRMM with diverse risk profiles and varying degrees of disease burden. Due to limited studies with head-to-head comparisons of BsAb in the treatment of RRMM, we performed direct and indirect comparisons between individual BsAbs and Standard of Care (SOC). Currently, most BsAb trials are single-arm monotherapy studies but what makes this analysis unique is the integration of real-world physicians’ choice data from global electronic health records, adjusted for baseline differences via IPTW and MAIC with clinical trial outcomes, making it the first of its kind comprehensive network meta-analysis by bridging these sources [53,58,59]. Apart from incorporating real-world physicians’ choice of care, this meta-analysis differs from a recently published meta-analysis as it includes both direct and indirect (network) pooled comparisons among different BsAbs and SOC, whereas the other meta-analysis was limited to just direct pooled analysis of few clinical trials [60].

Common challenges that occur during the treatment of RRMM usually erupt due to resistance mechanisms like T-cell fatigue or antigen escape; limiting the efficiency of single-epitope targeting therapies. Mechanisms like biallelic *TNFRSF17* (*BCMA*), *GPRC5D* deletions, and non-truncating *BCMA* ectodomain in-frame/missense mutations disrupt TCE binding, leading to preferential selection of *BCMA*-negative and *GPRC5D*-negative clones, with marked downregulation of these receptors, eventually contributing to antigen escape and limiting the efficacy of single-epitope-targeting therapies [7,61]. The findings of our analysis support antigen-switching strategies in RRMM. For ectodomain mutations impairing a *BCMA*-targeted TCE, switching to a non-cross-reactive construct (e.g., Teclistamab to Elranatamab with equivalent efficacy, ORR: 61% vs. 56%) remains a viable option. However, biallelic *BCMA* loss necessitates targeting alternative antigens like *GPRC5D* for optimal efficacy. This approach is substantiated by comparable ORR (68% vs. 61%) and ≥VGPR rates (37% vs. 47%) between *GPRC5D*-targeting Talquetamab and *BCMA*-targeting Teclistamab. Apart from antigen escape, T-cell fitness is an important parameter which measures how well T cells can proliferate, resist exhaustion, and sustain effector function and has emerged as a critical determinant of response to T-cell redirecting therapies. Patients whose CD8+ T-cell clones are more exhausted show impaired proliferation, reduced effector cytokine production and cytotoxicity. They also experience higher levels of inhibitory receptors (e.g., *PD-1*, *LAG-3*) and tend to have higher nonresponse rates and shorter PFS with *BCMA*-directed bispecifics. This underscores that pretreatment T-cell fitness strongly determines T-cell engager efficacy and toxicity [62]. Another component which is responsible for the potency and selectivity of T-cell engagers is the antigen density which is the number of target molecules per tumor cell and their spatial arrangement. Higher antigen density increases T-cell recruitment and tumor lysis but also increases the risk of on-target off-tumor effects. Studies have shown that tuning sensitivity to antigen density can preserve antitumor activity while limiting normal-tissue recognition [63].

Also, high disease burden, which is measured by tumor mass, circulating tumor cells, extramedullary disease, and aggressive tumor biology, contributes to both resistance and toxicity with T-cell engagers. This is because heavily infiltrated sites need strong T-cell infiltration and may favor immune escape, while large antigen load amplifies cytokine release and systemic inflammation. Tumor microenvironment, including immunosuppressive myeloid regulatory cells, regulatory T-cells, inhibitory cytokines, and metabolic constraints, has shown to blunt T-cell engager activity by reducing T-cell functionality and promoting exhaustion, whereas more permissive microenvironments support durable responses. Thus, these are classified as key “tumor-extrinsic” resistance mechanisms to TCEs [64,65].

During T-cell therapy, activated T cells and inflammatory mediators affect non-target cells. This “bystander” activation contributes to systemic cytokine-mediated toxicity and potential off-tumor tissue injury in organs expressing low levels of the target antigen. Therefore, organ-specific toxicity should be considered as part of safety assessment and target selection in current and future T-cell engager strategies [66,67]. In our analysis, patients who received Talquetamab experienced lower incidence of infections as compared to Teclistamab and Elranatamab (58% vs. 66% and 60%). Notably, the higher incidence of ≥grade 3 infections with Teclistamab and Elranatamab compared to Talquetamab (27% vs. 34% vs. 22%) parallels their greater rates of ≥grade 3 neutropenia (50% and 56% vs. 30%), suggesting that deeper treatment-related neutropenia with *BCMA*-directed bispecifics contributes to the excess burden of serious infections relative to *GPRC5D*-targeting agents. This can be attributed to the greater cytokine storm and more extensive depletion of the plasma-cell compartment, humoral immunity, marrow reserve, and hypogammaglobulinemia caused by *BCMA*-targeting agents than *GPRC5D*-targeting agents [68,69]. These observed differences in infection rates in our analysis are quantitative pooling of single-arm studies and their potential biological mechanisms are speculative and should be validated in prospective, head-to-head studies. A descriptive study from the REISAMIC registry reported that infectious complications with T-cell engagers occurred predominantly in heavily pretreated hematologic malignancies, with an infection-related mortality of 2.8%, highlighting pneumonia, COVID-19, and bacterial bloodstream infections as major contributors [70]. The Blood Cancer Journal expert consensus on monitoring, prophylaxis, and treatment of infections in bispecific-treated RRMM recommends HSV/VZV and PJP prophylaxis for all patients, but no routine antifungal prophylaxis except in high-risk or profoundly neutropenic patients. They also suggest use of granulocyte colony-stimulating factors for grade 3 neutropenia, and IVIG replacement to reduce serious infections, which is supported by observational data showing marked reductions in severe infections with IVIG in anti-*BCMA* bispecific recipients [71,72,73].

Other hematological side effects were quite similar between Talquetamab and Teclistamab with (31% vs. 24%) of >grade 3 anemia and 25% vs. 19% of >grade 3 thrombocytopenia. Also, Talquetamab and Teclistamab showed similar rates of CRS (68% vs. 66%) and a slightly higher incidence of CRS was observed with Elranatamab (74%). Higher incidence of ICANS was seen with *GPRC5D*-targeting agent Talquetamab (25%) as compared to *BCMA*-targeting agents Elranatamab and Teclistamab (11% and 7%). Moreover, dysgeusia, which is unique to *GPRC5D*-targeting agents, occurred in nearly 70% of patients in our analysis but was low grade and manageable. This is because *GPRC5D*-targeting agents engage T cells in tissue environments that are more prone to local inflammation and neural/vascular irritation and keratinized tissues, whereas *BCMA*-targeting agents predominantly attack the T cells within the lymphoid/plasma cell compartments.

Interestingly, our study also concludes that single-arm analysis of either *GPRC5D*-targeting monotherapy (e.g., Talquetamab) or *BCMA* alone (e.g., Teclistamab) have shown inferior ORR, ≥CR as compared to the pooled ORR of trials employing both *GPRC5D* and Daratumumab combination therapy. This could be attributed to Daratumumab’s ability to combat T-cell exhaustion due to its speculated ability of T-cell alteration through expansion of CD4+ helper T cells and CD8+ cytotoxic T cells in blood and marrow, causing a shift from naive to effector-memory CD8+ T cells which ultimately leads to release from an exhausted phenotype [74,75,76].

Moreover, the employment of Talquetamab and Teclistamab together showed superior ORRs than either Talquetamab or Teclistamab alone which is indicated by our analysis (86% with combination vs. 68% with Talquetamab and 61% with Teclistamab) and ≥VGPR (78% vs. 37% vs. 47%) with no substantial difference in CRS (79% vs. 68% vs. 61%) or infections (63% vs. 58% vs. 66%). While these findings suggest that dual targeting of *BCMA* and *GPRC5D* may enhance efficacy and potentially mitigate antigen escape, these observations are descriptive and hypothesis-generating. Given the heterogeneity of included studies and lack of direct randomized comparisons, these results should be interpreted with caution and warrant validation in prospective trials, such as those evaluating combination strategies (e.g., RedirecTT trial) [77].

### Strengths and Limitations

One of the major strengths of the study is the inclusion of heterogeneous populations across different geographical regions and a good blend of real-world data. Although most of the therapies approved for RRMM are based on single-arm clinical trials, analyses like ours can be useful to contextualize the clinical efficacy of novel agents against the real-world options and inform clinical decision-making. Although the application of IPTW and MAIC used to compare clinical trials with real-world data mitigated the effects of confounding and baseline covariate differences to a certain extent, we performed subgroup analysis on the basis of prior *BCMA* exposure but the real-world data was not adjusted for some parameters like cytogenetic risk, presence of extra medullary disease (EMD), and ECOG performance status, as all the factors were not reported uniformly in the real-world studies. This includes some inevitable “unmeasured confounding” bias which potentially includes either overestimation (or underestimation) of treatment effects. Secondly, patients without sufficient information in their electronic health records were excluded in the final inclusion in real-world data, introducing potential selection bias. Lastly, in the myeloma microenvironment, upregulation of *PD-1* on T-cells and *PD-L1* on malignant plasma cells contributes to T-cell anergy which hampers the cytotoxic activity of T-cell-redirecting BsAbs. Dual *PD-1*/*TIGIT* blockade has been shown to restore oxidative phosphorylation in exhausted T cells, enhancing BsAb or CAR-T efficacy in multiple myeloma models [78,79]. So, novel constructs such as *BCMA/GPRC5D × PDL-1* bispecific IgG1 molecules might show potent activity by simultaneously engaging tumor cells and locally inhibiting *PD-L1* signaling, which can be explored as a future treatment strategy in RRMM.

## 5. Conclusions

This network meta-analysis concludes that bispecific T-cell engagers that target *BCMA*, *GPRC5D*, *or FcRH5* provide significantly greater objective response rates as compared to SOC with comparable efficacy among them in RRMM. We also concluded that while a PFS benefit was observed in *BCMA*-naive patients with bispecific antibodies when compared to SOC, the effect is heterogeneous in *BCMA*-exposed populations and should be interpreted with caution and should be considered as hypothesis-generating, pending validation in prospective, subgroup-specific analyses. Our study concluded longer OS results with all bispecific agents as compared to SOC but most of the included studies had a median follow-up of approximately a year or considerable censoring at 12 months. This might have reduced the precision of 1-year Kaplan–Meier overall survival estimates which made our findings vulnerable to limitations related to follow-up duration and censoring, residual confounding, and post-treatment course. Because progression-free survival 2 (PFS2) and subsequent therapy details were not reported, we could not evaluate the impact of treatment sequencing and post-progression survival on OS. So, these findings are hypothesis-generating and should be interpreted cautiously in the absence of head-to-head randomized data. As a result, our conclusions should not be interpreted as definitive evidence that specific bispecific antibodies improve or worsen survival as compared with SOC, but rather as comparative efficacy signals that warrant confirmation in prospective randomized or pragmatic trials with head-to head comparisons.

## Figures and Tables

**Table 1:** Characteristics of single-arm trials and retrospective studies. Total population (n) denotes the entire treated cohort for each study and triple refractory (n) and penta-refractory population (n) denotes the absolute number of triple refractory and penta-refractory patients in each study. * In TRIMM-2 (NCT04108195), triple-class refractory status was reported only for the overall cohort (40/65 patients, 61.5%) and not by individual arm.

Study Name	Study Type	Total Population (n)	Triple Refractory Population (n)	Penta-Refractory Population (n)	Target	Prior LOT (*n*)	Median Follow-Up (Months)
Chari et al. 2025 (TRIMM-2 0.4 mg/kg QW) NCT04108195 [22]	Phase 1b	14	* (overall TRIMM-2 40/65, 61.5%)	NR	*GPRC5D*, *CD3*, *CD38*	5	18.6
Chari et al. 2025 (TRIMM-2 0.8 mg/kg Q2W) NCT04108195 [22]	Phase 1b	50	* (overall TRIMM-2 40/65, 61.5%)	NR	*GPRC5D*, *CD3*, *CD38*	5	18.6
Chari et al. 2025 (MonumenTAL-1 0.4 mg/kg QW) NCT04634552 [14]	Phase 2	143	106	42	*GPRC5D*, *CD3*	5	14.9
Chari et al. 2025 (MonumenTAL-1 0.8 mg/kg Q2W) NCT04634552 [14]	Phase 2	154	100	34	*GPRC5D*, *CD3*	4	31.2
Rasche et al. 2025 (MonumenTAL-1 (post–T-cell-redirected cohorts) NCT04634552 [23]	Phase 2	78	NR	NR	*GPRC5D*, *CD3*	≥3	16.8
Searle et al. 2024 (MonumenTAL-2 Cohort E) NCT05050097 [24]	Phase 1b	35	7	NR	*GPRC5D*, *CD3*	3	11.4
Popat et al. 2025 [25]	Retrospective	93	91	80	*GPRC5D*, *CD3*	5	15
Pasvolsky et al. 2025 [26]	Retrospective	484	NR	242	*GPRC5D*, *CD3*	6	N/A
Frenking et al. 2025 [27]	Retrospective	138	118	64	*GPRC5D*, *CD3*	6	8.2
Al hadidi et al. 2025 [28]	Retrospective	114	114	90	*GPRC5D*, *CD3*	6	10
Dhakal et al. 2024 [29]	Retrospective	77	56	NR	*GPRC5D*, *CD3*	4	N/A
Gill et al. 2024 [30]	Retrospective	27	26	NR	*GPRC5D*, *CD3*	8	N/A
Neupane et al. 2025 [31]	Retrospective	87	76	NR	*GPRC5D*, *CD3*	6	N/A
Donk et al. 2023 (MajesTEC-1 Cohort A, anti-*BCMA* naive) NCT04557098 [32]	Phase 2	165	68	NR	*BCMA*, *CD3*	5	30
Touzeau et al. 2024 (MajesTEC-1 Cohort C, prior anti-*BCMA*) NCT04557098 [15]	Phase 2	40	40	32	*BCMA*, *CD3*	5	28
D’Souza et al. 2024 (MajesTEC-2 Cohort A) NCT04722146 [33]	Phase 1b	17	9	0	*BCMA*, *CD3*, *CD38*	2	16.2
Offner et al. 2023 (MajesTEC-2 Cohort C) NCT04722146 [34]	Phase 1b	28	28	0	*BCMA*, *CD3*	4	14.7
Searle et al. 2023 (MajesTEC-2 Cohort E) NCT04722146 [35]	Phase 1b	32	15	NR	*BCMA*, *CD3*, *CD38*	2	8.4
Uttervall et al. 2026 [36]	Retrospective	113	89	50	*BCMA*, *CD3*	6	20.7
Tan et al. 2025 [37]	Retrospective	210	174	92	*BCMA*, *CD3*	6	5.3
Perrot et al. 2024 [38]	Retrospective	303	129	NR	*BCMA*, *CD3*	5	11.9
Reidhammer et al. 2024 [39]	Retrospective	123	114	74	*BCMA*, *CD3*	6	5.5
Mohan et al. 2024 [40]	Retrospective	110	95	84	*BCMA*, *CD3*	6	3.2
Dima et al. 2023 [41]	Retrospective	108	99	68	*BCMA*, *CD3*	5	3.8
Bahlis et al. 2023 (MagnetisMM-1) NCT03269136 [42]	Phase 1	55	50	43	*BCMA*, *CD3*	5	12
Lesokhin et al. 2023 (MagnetisMM-3 Cohort A *BCMA*-naïve) NCT04649359 [13]	Phase 2	123	119	52	*BCMA*, *CD3*	5	14.7
Lesokhin et al. 2023 (MagnetisMM-3 Cohort B, Prior *BCMA*) NCT04649359 [13]	Phase 2	63	61	27	*BCMA*, *CD3*	5	10.4
Malard et al. 2024 [43]	Retrospective	101	97	77	*BCMA*, *CD3*	5	15.5
Portuguese et al. 2025 [44]	Retrospective	125	118	61	*BCMA*, *CD3*	6	7.5
Lee et al. 2024 (LINKER-MM1 50 mg cohort) NCT03761108 [45]	Phase 1/2	104	97	56	*BCMA*, *CD3*	6	7.7
Bumma et al. 2024 (LINKER-MM1 200 mg cohort) NCT03761108 [46]	Phase 1/2	117	96	33	*BCMA*, *CD3*	5	21.3
Kumar et al. 2024 (CAMMA-2 A1) NCT05535244 [47]	Phase 1/2	21	21	NR	*FcRH5*, *CD3*	6	11
Ho et al. 2025 (CAMMA-3) NCT04910568 [48]	Phase 1	52	52	NR	*FcRH5*, *CD3*	5	6.5
Richter et al. 2024 (GO39775) NCT03275103 [49]	Phase 1	167	160	123	*FcRH5*, *CD3*	6	11.3
Harrison et al. 2025(CAMMA-1 Arm B) NCT04910568 [50]	Phase 1b	64	64	NR	*FcRH5*, *CD3*	2	N/A
Deforge et al. 2024 (CAMMA-2 A2) NCT05535244 [51]	Phase 1/2	21	18	13	*FcRH5*, *CD3*	8	5.7

**Table 2:** Characteristics of comparative studies. BsAb: Bispecific antibody, SOC: Standard of Care. MAIC: Matching-adjusted indirect comparison cohorts, IPTW: Inverse probability of treatment weighting comparison studies. NR: Not reported.

Study Name	Bispecific Antibody	Control	BsAb (*n*)	SOC (*n*)	BsAb Triple/Penta-Refractory (n)	SOC Triple/Penta-Refractory (n)	Study Type	Prior LOT BsAb (n)	Prior LOT Control (n)	Median Follow-Up, BsAb (in Months)	Median Follow-Up, SOC (in Months)
Mol et al. 2025 [52]	Elranatamab	SOC	123	248	123/NR	248/NR	MAIC	NR	NR	28.4	26.4
Costa et al. 2024-Flatiron Health [53]	Elranatamab	SOC	123	152	44/NR	36/NR	MAIC	5.2	4.0	15	N/A
Costa et al. 2024-COTA Comparisons [53]	Elranatamab	SOC	123	239	40/NR	59/NR	MAIC	5.2	4.9	15	N/A
Tsang et al. 2025 [54]	Elranatamab	SOC	123	81	123/NR	81/NR	IPTW	5	4	28.4	N/A
Kumar et al. 2024 [55]	Linvoseltamab	SOC	105	307	105/NR	307/NR	MAIC	NR	NR	14.3	N/A
Einsele et al. 2024 [56]	Talquetamab	SOC	145	177	145/NR	177/NR	MAIC	3	3	29.8	26.4
Ye et al. 2024 [57]	Talquetamab	SOC	143	1169	64/42	458/349	IPTW	NR	3.6	18.8	13.4
Krishnan et al. 2023 [58]	Teclistamab	SOC	165	364	78/50	169/119	MAIC	NR	NR	14.1	18.2
Moreau et al. 2024-LocoMMotion [59]	Teclistamab	SOC	165	248	20/50	30/93	MAIC	NR	NR	22.8	26.4
Moreau et al. 2024- LocoMMotion+ MoMMent pooled [59]	Teclistamab	SOC	165	302	20/50	37/108	MAIC	NR	NR	22.8	24.3

## Data Availability

No new data were created in this study. All data supporting the reported results are available within the article and its Supplementary File, and were derived from publicly available published studies cited, which are cited in the manuscript.

## Supplementary Materials

The following supporting information can be downloaded at: https://www.mdpi.com/article/10.3390/ijms27167179/s1.

## Abbreviations

RRRM	Relapsed/Refractory Multiple Myeloma.
PIs	Proteasome Inhibitors.
IMiDs	Immunomodulatory Drugs.
TCR	Triple-class refractory.
TCE	Triple-class exposed.
Ide-cel	Idecabtagene vicleucel.
Cilta-cel	Ciltacabtagene autoleucel.
BsAbs	Bispecific antibodies.
*BCMA*	B-cell maturation antigen.
*GPRC5D*	G protein-coupled receptor family C group 5 member D.
*FcRH5*	Fc receptor homolog 5.
vs.	versus.
PRISMA	Preferred Items for Systematic reviews and Meta-analyses.
PICOS	Patients, Interventions, Comparators, Outcomes, and Study design.
SOC	Standard of care.
ORR	Objective response rate.
VGPR	Very Good Partial Response.
CR	Complete Response.
CRS	Cytokine release syndrome.
PFS	Progression free survival.
OS	Overall survival.
ICANS	Immune Effector Cell-associated neurotoxicity syndrome.
NOS	Newcastle-Ottawa scale.
MAIC	Matching adjusted indirect comparison cohorts.
IPTW	Inverse probability of treatment weighting comparison studies.
CI	Confidence Interval.
OR	Odds Ratio.
HR	Hazard Ratio.
EMD	Extra medullary disease.
ECOG	Eastern Cooperative Oncology Group.
*PD-1*	Programmed Cell Death Protein 1.
*TIGIT*	T Cell Immunoreceptor with Ig and ITIM Domains.

## References

[1] Kumar S., Paiva B., Anderson K.C., Durie B., Landgren O., Moreau P., Munshi N., Lonial S., Bladé J., Mateos M.-V. (2016). International Myeloma Working Group consensus criteria for response and minimal residual disease assessment in multiple myeloma. Lancet Oncol..

[2] Moreau P., Kumar S.K., San Miguel J., Davies F., Zamagni E., Bahlis N., Ludwig H., Mikhael J., Terpos E., Schjesvold F. (2021). Treatment of relapsed and refractory multiple myeloma: Recommendations from the International Myeloma Working Group. Lancet Oncol..

[3] Costa L.J., Hungria V., Mohty M., Mateos M. (2022). How I treat TRIPLE-CLASS refractory multiple myeloma. Br. J. Haematol..

[4] Mateos M.V., Weisel K., De Stefano V., Goldschmidt H., Delforge M., Mohty M., Dytfeld D., Angelucci E., Vincent L., Perrot A. (2024). LocoMMotion: A study of real-life current standards of care in triple-class exposed patients with relapsed/refractory multiple myeloma—2-year follow-up (final analysis). Leukemia.

[5] Munshi N.C., Anderson L.D., Shah N., Madduri D., Berdeja J., Lonial S., Raje N., Lin Y., Siegel D., Oriol A. (2021). Idecabtagene Vicleucel in Relapsed and Refractory Multiple Myeloma. N. Engl. J. Med..

[6] Jagannath S., Martin T.G., Lin Y., Cohen A.D., Raje N., Htut M., Deol A., Agha M., Berdeja J.G., Lesokhin A.M. (2025). Long-Term (≥5-Year) Remission and Survival After Treatment With Ciltacabtagene Autoleucel in CARTITUDE-1 Patients With Relapsed/Refractory Multiple Myeloma. J. Clin. Oncol..

[7] Lee H., Ahn S., Maity R., Leblay N., Ziccheddu B., Truger M., Chojnacka M., Cirrincione A., Durante M., Tilmont R. (2023). Mechanisms of antigen escape from *BCMA*- or *GPRC5D*-targeted immunotherapies in multiple myeloma. Nat. Med..

[8] Reyes K.R., Liu Y.C., Huang C.Y., Banerjee R., Martin T., Wong S.W., Wolf J.L., Arora S., Shah N., Chari A. (2024). Salvage therapies including retreatment with *BCMA*-directed approaches after *BCMA* CAR-T relapses for multiple myeloma. Blood Adv..

[9] Alqazaqi R., Schinke C., Thanendrarajan S., Zangari M., Shaughnessy J., Zhan F., Tricot G., Van Rhee F., Al Hadidi S. (2022). Geographic and Racial Disparities in Access to Chimeric Antigen Receptor–T Cells and Bispecific Antibodies Trials for Multiple Myeloma. JAMA Netw. Open.

[10] Peres L.C., Oswald L.B., Dillard C.M., De Avila G., Nishihori T., Blue B.J., Freeman C.L., Locke F.L., Alsina M., Puglianini O.C. (2024). Racial and ethnic differences in clinical outcomes among patients with multiple myeloma treated with CAR T-cell therapy. Blood Adv..

[11] Bhutani M., Habib A., Vegel A., De Avila G., Nishihori T., Blue B.J., Freeman C.L., Locke F.L., Alsina M., Castaneda Puglianini O. (2025). Outcomes of CAR T-Cell therapy in relapsed/refractory multiple myeloma by race: A multicenter real-world study. Blood Cancer J..

[12] Moreau P., Garfall A.L., Van De Donk N.W.C.J., Nahi H., San-Miguel J.F., Oriol A., Nooka A.K., Martin T., Rosinol L., Chari A. (2022). Teclistamab in Relapsed or Refractory Multiple Myeloma. N. Engl. J. Med..

[13] Lesokhin A.M., Tomasson M.H., Arnulf B., Bahlis N.J., Miles Prince H., Niesvizky R., Rodrίguez-Otero P., Martinez-Lopez J., Koehne G., Touzeau C. (2023). Elranatamab in relapsed or refractory multiple myeloma: Phase 2 MagnetisMM-3 trial results. Nat. Med..

[14] Chari A., Touzeau C., Schinke C., Minnema M.C., Berdeja J.G., Oriol A., Van De Donk N.W.C.J., Rodríguez-Otero P., Morillo D., Martinez-Chamorro C. (2025). Safety and activity of talquetamab in patients with relapsed or refractory multiple myeloma (MonumenTAL-1): A multicentre, open-label, phase 1–2 study. Lancet Haematol..

[15] Touzeau C., Krishnan A.Y., Moreau P., Perrot A., Usmani S.Z., Manier S., Cavo M., Martinez Chamorro C., Nooka A.K., Martin T.G. (2024). Efficacy and safety of teclistamab in patients with relapsed/refractory multiple myeloma after *BCMA*-targeting therapies. Blood.

[16] Fandrei D., Seiffert S., Rade M., Rieprecht S., Gagelmann N., Born P., Wiemers T., Weidner H., Kreuz M., Schassberger T. (2025). Bispecific Antibodies as Bridging to *BCMA* CAR-T Cell Therapy for Relapsed/Refractory Multiple Myeloma. Blood Cancer Discov..

[17] Page M.J., McKenzie J.E., Bossuyt P.M., Boutron I., Hoffmann T.C., Mulrow C.D., Shamseer L., Tetzlaff J.M., Akl E.A., Brennan S.E. (2021). The PRISMA 2020 statement: An updated guideline for reporting systematic reviews. BMJ.

[18] Higgins J.P.T., Altman D.G., Gøtzsche P.C., Jüni P., Moher D., Oxman A.D., Savovic J., Schulz K.F., Weeks L., Sterne J.A.C. (2011). The Cochrane Collaboration’s tool for assessing risk of bias in randomised trials. BMJ.

[19] DerSimonian R., Laird N. (1986). Meta-analysis in clinical trials. Control Clin. Trials.

[20] Stang A. (2010). Critical evaluation of the Newcastle-Ottawa scale for the assessment of the quality of nonrandomized studies in meta-analyses. Eur. J. Epidemiol..

[21] Sterne J.A.C., Hernán M.A., Reeves B.C., Savović J., Berkman N.D., Viswanathan M., Henry D., Altman D.G., Ansari M.T., Boutron I. (2016). ROBINS-I: A tool for assessing risk of bias in non-randomised studies of interventions. BMJ.

[22] Chari A., van de Donk N.W.C.J., Dholaria B., Weisel K., Mateos M.-V., Goldschmidt H., Martin T.G., Morillo D., Reece D., Rodríguez-Otero P. (2025). Talquetamab plus daratumumab for the treatment of relapsed or refractory multiple myeloma in the TRIMM-2 study. Blood.

[23] Rasche L., Schinke C.D., Touzeau C., Minnema M., Van De Donk N.W., Rodríguez-Otero P., Mateos M.-V., Ye J.C., Tomlinson C., Vishwamitra D. (2025). Efficacy and safety from the phase 1/2 MonumenTAL-1 study of talquetamab, a *GPRC5D×CD3* bispecific antibody, in patients with relapsed/refractory multiple myeloma: Analyses at an extended median follow-up. J. Clin. Oncol..

[24] Searle E., Quach H., Biran N., Perrot A., Berdeja J.G., Dorritie K., Van Elssen C., Touzeau C., Anguille S., Vishwamitra D. (2024). MM-349 Talquetamab (Tal), a *GPRC5D × CD3* Bispecific Antibody (BsAb), in Combination With Pomalidomide (Pom) in Patients With Relapsed/Refractory Multiple Myeloma (RRMM): Efficacy and Safety Results From the Phase 1b MonumenTAL-2 Study. Clin. Lymphoma Myeloma Leuk..

[25] Popat R., Uttervall K., Perrot A., Magen H., Mykytiv V., Liberatore C., Zamagni E., Da Vià M.C., Antonioli E., Hansson M. (2025). Safety results from REALiTAL: A multi-country observational retrospective study of talquetamab in patients with relapsed/refractory multiple myeloma outside of clinical trials. Blood.

[26] Pasvolsky O., Grajales-Cruz A., Hansen D., Feng L., Dima D., Afrough A., Shain K., Banerjee R., Zanwar S., Anwer F. (2025). Talquetamab for relapsed/refractory multiple myeloma: Real-world outcomes from the US multiple myeloma immunotherapy consortium. Blood.

[27] Frenking J.H., Riedhammer C., Teipel R., Bassermann F., Besemer B., Bewarder M., Braune J., Brioli A., Brunner F., Dampmann M. (2025). A German multicenter real-world analysis of talquetamab in 138 patients with relapsed/refractory multiple myeloma. HemaSphere.

[28] Al Hadidi S., Szabo A., Mohan Lal B., Bag A., Zhen S., Ogunsesan Y., Annyapu E., Dhakal B., Bhutani D., Thanendrarajan S. (2025). Talquetamab in relapsed refractory multiple myeloma: Multi-institutional real-world study. Blood Cancer J..

[29] Dhakal B., Akhtar O.S., Cowan A.J., Richard S., Friend R., Rees M.J., Costello P., Vazquez Martinez M., Pasvolsky O., Wagner C.B. (2024). Talquetamab Bridging: Paving the Way to B-Cell Maturation Antigen (*BCMA*) CAR-T Cell Therapy in Relapsed/Refractory Multiple Myeloma (RRMM). Blood.

[30] Gill S.K., Fleming E., Gebre H., Bangolo A.I., Siegel D.S., Vesole D.H., Biran N., Parmar H., Phull P. (2024). Real World Outcomes with Talquetamab, a T-Cell-Redirecting *GPRC5D* Bispecific Antibody: A Single Center Experience for Relapsed/Refractory Multiple Myeloma (RRMM). Blood.

[31] Neupane K., Wright C., Whiting J., Harada K., De Avila G., Peres L., Vazquez-Martinez M., Blue B., Fadul J., Dixon A. (2025). Real-world outcomes of talquetamab in Relapsed/Refractory multiple myeloma. Blood.

[32] Van De Donk N.W.C.J., Moreau P., Garfall A.L., Bhutani M., Oriol A., Nooka A.K., Martin T.G., Rosiñol L., Mateos M.-V., Bahlis N.J. (2023). Long-term follow-up from MajesTEC-1 of teclistamab, a B-cell maturation antigen (*BCMA*) × *CD3* bispecific antibody, in patients with relapsed/refractory multiple myeloma (RRMM). J. Clin. Oncol..

[33] D’Souza A., Costa L.J., San-Miguel J.F., Berdeja J.G., Giles D.M., Touzeau C., McKay J.T., Dholaria B., Martin T.G., Perrot A. (2024). Teclistamab, Daratumumab, and Pomalidomide in Patients with Relapsed/Refractory Multiple Myeloma: Results from the Majestec-2 Cohort a and Trimm-2 Studies. Blood.

[34] Offner F., Decaux O., Hulin C., Anguille S., Sophie Michallet A., Costa L., Touzeau C., Boyd K., Vishwamitra D., Guo Y. (2023). S194: Teclistamab (TEC) + nirogacestat (NIRO) in relapsed/refractory multiple myeloma (RRMM): The phase 1b MAJESTEC-2 study. HemaSphere.

[35] Searle E., Quach H., Wong S., Costa L., Hulin C., Janowski W., Berdeja J., Anguille S., Matous J., Touzeau C. (2023). P30 single cohort results from majestec-2: Teclistamab (TEC) in combination with subcutaneous daratumumab (dara) and lenalidomide (LEN) in patients with multiple myeloma (MM). HemaSphere.

[36] Uttervall K., Kortum M.K., Perrot A., Farmer S.L., Cavo M., Kishore B., Jacquet C., Casanova M., Hansson M., Weisel K. (2026). REALiTEC: A multi-country observational retrospective study of teclistamab in patients with relapsed/refractory multiple myeloma outside of clinical trials. Haematologica.

[37] Tan C.R., Asoori S., Huang C.Y., Brunaldi L., Popat R., Kastritis E., Martinez-Lopez J., Bansal R., De Menezes Silva Corraes A., Chhabra S. (2025). Real-world evaluation of teclistamab for the treatment of relapsed/refractory multiple myeloma (RRMM): An International Myeloma Working Group Study. Blood Cancer J..

[38] Perrot A., Hulin C., Boumendil A., Manjra H., Leveque A., Croizier C., Dony A., Mohty M., Roussel M., Manier S. (2024). Teclistamab in relapsed refractory multiple myeloma: A multi-institutional real-world study from the French early access program. Haematologica.

[39] Riedhammer C., Bassermann F., Besemer B., Bewarder M., Brunner F., Carpinteiro A., Einsele H., Faltin J., Frenking J., Gezer D. (2024). Real-world analysis of teclistamab in 123 RRMM patients from Germany. Leukemia.

[40] Mohan M., Monge J., Shah N., Luan D., Forsberg M., Bhatlapenumarthi V., Balev M., Patwari A., Cheruvalath H., Bhutani D. (2024). Teclistamab in relapsed refractory multiple myeloma: Multi-institutional real-world study. Blood Cancer J..

[41] Dima D., Davis J.A., Ahmed N., Jia X., Sannareddy A., Shaikh H., Shune L., Kaur G., Khouri J., Afrough A. (2024). Safety and Efficacy of Teclistamab in Patients with Relapsed/Refractory Multiple Myeloma: A Real-World Experience. Transplant. Cell. Ther..

[42] Bahlis N.J., Costello C.L., Raje N.S., Levy M.Y., Dholaria B., Solh M., Tomasson M.H., Damore M.A., Jiang S., Basu C. (2023). Elranatamab in relapsed or refractorymultiple myeloma: The MagnetisMM-1 phase 1 trial. Nat. Med..

[43] Malard F., Bobin A., Labopin M., Karlin L., Frenzel L., Roussel M., Vignon M., Godet S., Chalopin T., Moyer P. (2024). Elranatamab monotherapy in the real-word setting in relapsed-refractory multiple myeloma: Results of the French compassionate use program on behalf of the IFM. Blood Cancer J..

[44] Portuguese A., Davis J., Raza S., Castaneda Puglianini O.A., Freeman C.L., Alsina M., Wright C., Goel U., Khouri J., Anwer F. (2025). Real-world outcomes with elranatamab in multiple myeloma: A multi-center analysis from the United States multiple myeloma immunotherapy consortium. Blood.

[45] Lee H.C., Bumma N., Richter J.R., Dhodapkar M.V., Hoffman J.E., Suvannasankha A., Zonder J.A., Shah M.R., Lentzsch S., Maly J.J. (2023). LINKER-MM1 study: Linvoseltamab (REGN5458) in patients with relapsed/refractory multiple myeloma. J. Clin. Oncol..

[46] Bumma N., Richter J., Jagannath S., Lee H.C., Hoffman J.E., Suvannasankha A., Zonder J.A., Shah M.R., Lentzsch S., Baz R. (2024). Linvoseltamab for Treatment of Relapsed/Refractory Multiple Myeloma. J. Clin. Oncol..

[47] Kumar S., Berdeja J., Sborov D., Corradini P., Cohen Y.C., Lesokhin A.M. Cevostamab in patients with RRMM who are triple-class refractory and have received a prior *BCMA*-targeted ADC or CAR T-cell: Initial results from the phase I/II CAMMA 2 study. Proceedings of the European Hematology Association (EHA) Annual Meeting.

[48] Ho P.J., Quach H., Delimpasi S., Santoro A., H-S Lee C., Kint N., Faict S., Yu Y., Catalani O., Mishra A. (2025). Subcutaneous cevostamab demonstrates manageable safety and clinically meaningful activity in Relapsed/Refractory multiple myeloma (RRMM): First results from the Phase Ib CAMMA 3 study. Blood.

[49] Richter J., Thomas S.K., Krishnan A.Y., Laubach J.P., Cohen A.D., Trudel S., Costa L.J., Bahlis N.J., Forsberg P.A., Kaedbey R. (2024). Cevostamab in Patients with Heavily Pretreated Relapsed/Refractory Multiple Myeloma (RRMM): Updated Results from an Ongoing Phase I Study Demonstrate Clinically Meaningful Activity and Manageable Safety and Inform the Doses and Regimen for Combination Studies. Blood.

[50] Harrison S., Riley C.H., Mian H., Mian H., Spencer A., Lavi N., Paris L., Mihalyova J., Popat R., Szarejko M. (2025). Tumor clearance, T-cell fitness, and minimal residual disease (MRD) outcomes in patients with relapsed/refractory multiple myeloma (RRMM) treated with cevostamab plus pomalidomide and dexamethasone: Biomarker analyses from CAMMA 1 Arm B. Blood.

[51] Delforge M., Richter J., Cohen Y.C., Corradini P., Sborov D.W., Lesokhin A.M., Berdeja J.G., Gatt M.E., Paris L., Rosinol Dachs L. (2024). Biomarker Correlates and Clinical Activity of Cevostamab in Patients (pts) with Triple-Class Refractory Multiple Myeloma (MM) Who Have Received ≥1 Prior B-Cell Maturation Antigen (*BCMA*)-Targeted Bispecific Antibody (BsAb): Results from the Phase I/II CAMMA 2 Study. Blood.

[52] Mol I., Hu Y., LeBlanc T.W., Cappelleri J.C., Chu H., Nador G., Aydin D., Cruz I.P., Hlavacek P. (2025). Elranatamab versus physician’s choice of treatment in patients with triple-class exposed/refractory multiple myeloma: An updated matching-adjusted indirect comparison. J. Comp. Eff. Res..

[53] Costa L.J., LeBlanc T.W., Tesch H., Sonneveld P., Kyle R.P., Sinyavskaya L., Hlavacek P., Meche A., Ren J., Schepart A. (2024). Elranatamab efficacy in MagnetisMM-3 compared with real-world control arms in triple-class refractory multiple myeloma. Future Oncol..

[54] Tsang C., O’Reilly J.E., Carpenter L., Duffield C., Tunaru F., Wallis J., Perkins A., Price T., Wood S., Ramasamy K. (2025). Comparison of outcomes with elranatamab and real world treatments in the UK for triple class exposed relapsed and refractory multiple myeloma. BMC Cancer.

[55] Kumar S., Jagannath S., Weisel K.C., Rosiñol Dachs L., Dimopoulos M.-A., Siegel D.S., Monge J., Leleu X., Du J., De La Rubia J. (2024). Comparative Effectiveness of Linvoseltamab Versus Current Real-World (RW) Standard-of-Care (SOC) Therapies in Triple-Class Exposed Relapsed/Refractory Multiple Myeloma (RRMM): Key Subgroups Analysis. Blood.

[56] Einsele H., Moreau P., Bahlis N., Bhutani M., Vincent L., Karlin L., Perrot A., Goldschmidt H., Van De Donk N.W.C.J., Ocio E.M. (2024). Comparative Efficacy of Talquetamab vs. Current Treatments in the LocoMMotion and MoMMent Studies in Patients with Triple-Class-Exposed Relapsed/Refractory Multiple Myeloma. Adv. Ther..

[57] Ye J.C., Biran N., Nair S., Lin X., Qi K., Londhe A., Ammann E., Renaud T., Kane C., Parekh T. (2025). Talquetamab Versus Real-World Physician’s Choice Treatment: Comparative Effectiveness in Patients With Triple-Class Exposed Relapsed/Refractory Multiple Myeloma. Clin. Lymphoma Myeloma Leuk..

[58] Krishnan A., Nooka A.K., Chari A., Garfall A.L., Martin T.G., Nair S., Lin X., Qi K., Londhe A., Pei L. (2023). Teclistamab versus real-world physician’s choice of therapy in triple-class exposed relapsed/refractory multiple myeloma. J. Comp. Eff. Res..

[59] Moreau P., Mateos M.V., Gonzalez Garcia M.E., Einsele H., De Stefano V., Karlin L., Lindsey-Hill J., Besemer B., Vincent L., Kirkpatrick S. (2024). Comparative Effectiveness of Teclistamab Versus Real-World Physician’s Choice of Therapy in LocoMMotion and MoMMent in Triple-Class Exposed Relapsed/Refractory Multiple Myeloma. Adv. Ther..

[60] Bakogeorgou S., Filippatos C., Malandrakis P., Tentolouris A., Terpos E., Gavriatopoulou M., Ntanasis-Stathopoulos I. (2025). Safety and Efficacy of Bispecific Antibody Treatment in Relapsed/Refractory Multiple Myeloma: A Systematic Review and Meta-Analysis of Proportions from Clinical Trials. Cancers.

[61] Lee H., Ahn S., Gonzales G.A., Leblay N., Barakat E., Greening D., Colarusso P., Canton J., Benaoudia S., Hasheminasabgorji E. (2026). Multimodal antigenic escape to *GPRC5D*-targeted T cell engagers in multiple myeloma. Nat. Med..

[62] Theprungsirikul P., Yu M., Rall K., Matthews M., Neparidze N., Parker T.L., Browning S., Anderson T., Stevens E., Foss F.M. (2024). Associations of T-cell fitness prior to B-cell maturation antigen (*BCMA*)–targeted chimeric antigen receptor T-cell (CART) and bispecific T-cell engager (BiTE) therapies and efficacy/toxicity in relapsed/refractory multiple myeloma (RRMM). J. Clin. Oncol..

[63] Bluemel C., Hausmann S., Fluhr P., Sriskandarajah M., Stallcup W.B., Baeuerle P.A., Kufer P. (2010). Epitope distance to the target cell membrane and antigen size determine the potency of T cell-mediated lysis by BiTE antibodies specific for a large melanoma surface antigen. Cancer Immunol. Immunother..

[64] Cao L., Leclercq-Cohen G., Klein C., Sorrentino A., Bacac M. (2025). Mechanistic insights into resistance mechanisms to T cell engagers. Front. Immunol..

[65] Baeuerle P.A., Sauer K., Grieshaber-Bouyer R., Michaelson J.S. (2026). T cell engagers emerge as a compelling therapeutic modality. J. Exp. Med..

[66] Yang Q., Wang S., Liu F., Yu Y., Zhao H. (2026). The trinity of T cell engagement: Navigating the molecular and clinical landscape of CAR-T, TILs, and TCEs in the war against cancer. Front. Immunol..

[67] Garcia-Lorenzo E., Dorta M., Doger B., Pedregal M., Moreno V. (2026). Landscape of T-cell engagers in solid tumors. Oncologist.

[68] Mohan M., Nagavally S., Dhakal B., Radhakrishnan S.V., Chhabra S., D’Souza A., Hari P. (2022). Risk of infections with B-cell maturation antigen-directed immunotherapy in multiple myeloma. Blood Adv..

[69] Hammons L.R., Szabo A., Janardan A., Dhakal B., Chhabra S., D’Souza A., Mohan M. (2022). Kinetics of Humoral Immunodeficiency With Bispecific Antibody Therapy in Relapsed Refractory Multiple Myeloma. JAMA Netw. Open.

[70] Hueso T., Cagnat J., Ouali K., Haberstich M., Henry B., Danu A., Bigenwald C., Merad M., Messayke S., Laparra A. (2025). Infectious Complications in Patients Treated With T-cell Engagers as Cancer Immunotherapies, a Descriptive Study From the REISAMIC Registry. Clin. Infect. Dis..

[71] Raje N., Anderson K., Einsele H., Efebera Y., Gay F., Hammond S.P., Lesokhin A.M., Lonial S., Ludwig H., Moreau P. (2023). Monitoring, prophylaxis, and treatment of infections in patients with MM receiving bispecific antibody therapy: Consensus recommendations from an expert panel. Blood Cancer J..

[72] Popat R., Cheok K., Sridhar A., Feely C., Mactier C., Mcmillan A., Wechalekar A.D., Rabin N., Sive J., Kyriakou C. (2023). Infection Risk and Use of Prophylaxis with Anti-*BCMA* Bispecific T Cell Engagers and Belantamab Mafodotin for Patients with Relapsed and Refractory Multiple Myeloma. Blood.

[73] Lancman G., Parsa K., Kotlarz K., Avery L., Lurie A., Lieberman-Cribbin A., Cho H.J., Parekh S.S., Richard S., Richter J. (2023). IVIg Use Associated with Ten-Fold Reduction of Serious Infections in Multiple Myeloma Patients Treated with Anti-*BCMA* Bispecific Antibodies. Blood Cancer Discov..

[74] Krejcik J., Casneuf T., Nijhof I.S., Verbist B., Bald J., Plesner T., Syed K., Liu K., Van De Donk N.W.C.J., Weiss B.M. (2016). Daratumumab depletes *CD38*^+^ immune regulatory cells, promotes T-cell expansion, and skews T-cell repertoire in multiple myeloma. Blood.

[75] Cerrano M., Castella B., Lia G., Olivi M., Faraci D.G., Butera S., Martella F., Scaldaferri M., Cattel F., Boccadoro M. (2020). Immunomodulatory and clinical effects of daratumumab in T-cell acute lymphoblastic leukaemia. Br. J. Haematol..

[76] Leblay N., Maity R., Hasan F., Neri P. (2020). Deregulation of Adaptive T Cell Immunity in Multiple Myeloma: Insights Into Mechanisms and Therapeutic Opportunities. Front. Oncol..

[77] Mateos M.V., Magen H., Gatt M., Sebag M., Kim K., Min C.-K., Ocio E., Yoon S.-S., Chu M., Rodriguez-Otero P. (2025). Safety and efficacy of talquetamab + teclistamab in patients with Relapsed/Refractory multiple myeloma from Phase 1b of redirectt-1: Results with an extended median follow-up of 3 years. Blood.

[78] Ouyang W., Jin S.W., Xu N., Liu W.-Y., Zhao H., Zhang L., Kang L., Tao Y., Liu Y., Wang Y. (2024). *PD-1* downregulation enhances CAR-T cell antitumor efficiency by preserving a cell memory phenotype and reducing exhaustion. J. Immunother. Cancer.

[79] Xia Y., Zhu J., Guo R., Shen X., Shi M., Shen N., Fan L., Chen L. (2025). Preclinical evaluation of *TIGIT* as a target to enhance efficacy and mitigate T cell exhaustion in multiple myeloma following *BCMA*-CAR-T therapy. Cell Death Dis..

