# Supplementary material for: T-Cell Engagers Targeting BCMA, GPRC5D, or FcRH5 in Relapsed/Refractory Multiple Myeloma: The Landscape Beyond CAR-T Cell Therapy-A Systematic Review and Network Meta-Analysis
Source: Int J Mol Sci. 2026 Aug 11;27(16):7179. doi: 10.3390/ijms27167179 (PMC13513919; doi:10.3390/ijms27167179)
Supplement: Supplementary file 1 [file ijms-27-07179-s001.zip › ijms-4332552-supplementary.pdf]

Supplementary file:

Database	Search String	Results
PubMed	("Multiple Myeloma"[Mesh] OR "Multiple Myeloma/drug therapy"[Mesh] OR "Neoplasms, Plasma Cell/drug therapy"[Mesh] OR "multiple myeloma"[Title/Abstract] OR "plasma cell myeloma"[Title/Abstract] OR "relapsed multiple myeloma"[Title/Abstract] OR "refractory multiple myeloma"[Title/Abstract]) AND ("bispecific antibody"[Title/Abstract] OR "bispecific antibodies"[Title/Abstract] OR "bispecific T-cell engager"[Title/Abstract] OR "T cell engager"[Title/Abstract] OR "BCMA bispecific"[Title/Abstract] OR "B-cell maturation antigen"[Title/Abstract] OR "GPRC5D"[Title/Abstract] OR talquetamab[Title/Abstract] OR teclistamab[Title/Abstract] OR elranatamab[Title/Abstract] OR linvoseltamab[Title/Abstract])	1669
Clinical Trials.gov	(“Multiple Myeloma” OR “Plasma Cell Neoplasm” OR “Plasma Cell Neoplasms” OR “Plasma Cell Myeloma” OR “Relapsed Multiple Myeloma” OR “Refractory Multiple Myeloma”) AND (“Bispecific Antibodies” OR “Bispecific Antibody” OR “bispecific T – cell engager” OR “BCMA bispecific” OR “B – cell maturation antigen” OR “GPRC5D” OR “Talquetamab” OR “Teclistamab” OR “Elranatamab” OR “Linvoseltamab”)	237
EMBASE	('multiple myeloma'/exp OR 'multiple myeloma' OR 'plasma cell neoplasm'/exp OR 'plasma cell neoplasm' OR 'plasma cell neoplasms'/exp OR 'plasma cell neoplasms' OR 'plasma cell myeloma'/exp OR 'plasma cell myeloma' OR 'relapsed multiple myeloma'/exp OR 'relapsed multiple myeloma' OR 'refractory multiple myeloma'/exp OR 'refractory multiple myeloma') AND ('bispecific antibodies' OR 'bispecific antibody'/exp OR 'bispecific antibody' OR 'bispecific t – cell engager' OR 'BCMA bispecific' OR 'b – cell maturation antigen' OR 'gprc5d' OR 'talquetamab'/exp OR 'talquetamab' OR 'teclistamab'/exp OR 'teclistamab' OR 'elranatamab'/exp OR 'elranatamab' OR 'linvoseltamab'/exp OR 'linvoseltamab')	3154
Scopus	(“Multiple Myeloma” OR “Plasma Cell Neoplasm” OR “Plasma Cell Neoplasms” OR “Plasma Cell Myeloma” OR “Relapsed Multiple Myeloma” OR “Refractory Multiple Myeloma”) AND (“Bispecific Antibodies” OR “Bispecific Antibody” OR “bispecific T – cell engager” OR “BCMA bispecific” OR “B – cell maturation antigen” OR “GPRC5D” OR “Talquetamab” OR “Teclistamab” OR “Elranatamab” OR “Linvoseltamab”)	2652

Web of Science	(“Multiple Myeloma” OR “Plasma Cell Neoplasm” OR “Plasma Cell Neoplasms” OR “Plasma Cell Myeloma” OR “Relapsed Multiple Myeloma” OR “Refractory Multiple Myeloma”) AND (“Bispecific Antibodies” OR “Bispecific Antibody” OR “bispecific T – cell engager” OR “BCMA bispecific” OR “B – cell maturation antigen” OR “GPRC5D” OR “Talquetamab” OR “Teclistamab” OR “Elranatamab” OR “Linvoseltamab”)	2850
CINAHL	(“Multiple Myeloma” OR “Plasma Cell Neoplasm” OR “Plasma Cell Neoplasms” OR “Plasma Cell Myeloma” OR “Relapsed Multiple Myeloma” OR “Refractory Multiple Myeloma”) AND (“Bispecific Antibodies” OR “Bispecific Antibody” OR “bispecific T – cell engager” OR “BCMA bispecific” OR “B – cell maturation antigen” OR “GPRC5D” OR “Talquetamab” OR “Teclistamab” OR “Elranatamab” OR “Linvoseltamab”)	294
Google Scholar	(“Multiple Myeloma” OR “Plasma Cell Neoplasm” OR “Plasma Cell Neoplasms” OR “Plasma Cell Myeloma” OR “Relapsed Multiple Myeloma” OR “Refractory Multiple Myeloma”) AND (“Bispecific Antibodies” OR “Bispecific Antibody” OR “bispecific T – cell engager” OR “BCMA bispecific” OR “B – cell maturation antigen” OR “GPRC5D” OR “Talquetamab” OR “Teclistamab” OR “Elranatamab” OR “Linvoseltamab”)	100

Table S1: Search Strings.

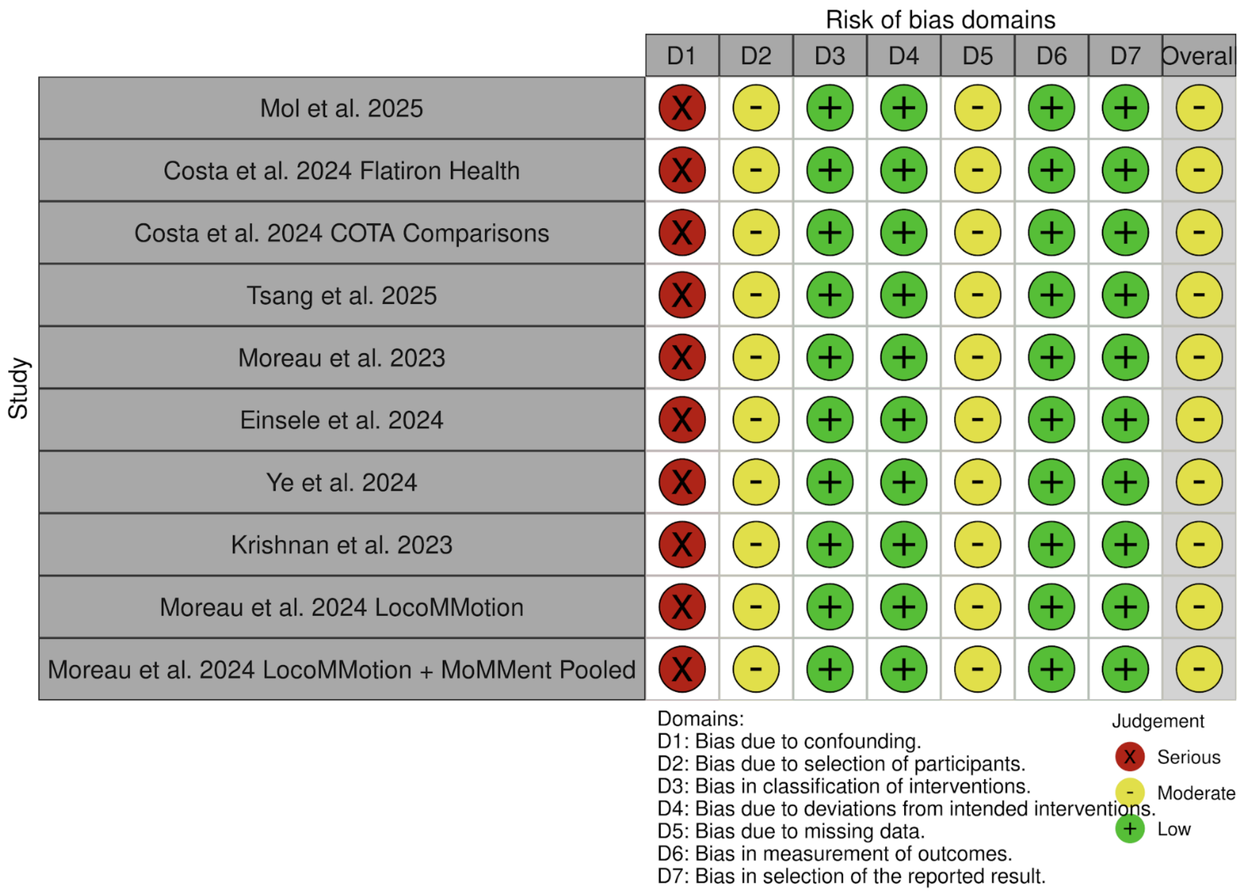

Figure S1: Risk of bias in the comparative studies.

Study	D1	D2	D3	D4	D5	D6	D7	Overal
Chari et al. 2025 (TRIMM-2 0.4 mg /kg QW)	X	-	+	+	-	+	-	-
Chari et al. 2025 (TRIMM-2 0.8 mg /kg Q2W)	X	-	+	+	-	+	-	-
Chari et al. 2025 (MonumenTAL-1 0.4 mg /kg QW)	X	-	+	+	-	+	-	-
Chari et al. 2025 (MonumenTAL-1 0.8 mg /kg Q2W)	X	X	+	+	-	+	-	X
Rasche et al. 2025	X	-	+	+	-	+	-	-
Searle et al. 2024	X	-	+	+	-	+	-	-
Donk et al. 2023	X	-	+	+	-	+	-	-
Touzeau et al. 2024	X	X	+	+	-	+	-	X
D'Souza et al. 2024	X	-	+	+	-	+	-	-
Offner et al. 2023	X	-	+	+	-	+	-	-
Searle et al. 2023	X	-	+	+	-	+	-	-
Bahlis et al. 2023	X	+	+	+	-	+	-	-
Lesokhin et al. 2023 MagnetisMM-3 Cohort A (BCMA-naïve)	X	+	+	+	-	+	-	-
Lesokhin et al. 2023 MagnetisMM-3 Cohort B (Prior BCMA)	X	+	+	+	-	+	-	-
Lee et al. 2024	X	+	+	+	-	+	-	-
Bumma et al. 2024	X	+	+	+	-	+	-	-
Ho et al. 2025	X	-	+	+	-	+	-	-
Richter et al. 2024	X	-	+	+	-	+	-	-
Harrison et al. 2025	X	-	+	+	-	+	-	-
Kumar et al. 2024	X	X	+	+	-	+	-	X
Delforge et al. 2024	X	X	+	+	-	+	-	X

Risk of bias domains

Domains:
D1: Bias due to confounding.
D2: Bias due to selection of participants.
D3: Bias in classification of interventions.
D4: Bias due to deviations from intended interventions.
D5: Bias due to missing data.
D6: Bias in measurement of outcomes.
D7: Bias in selection of the reported result.

Judgement
X Serious
- Moderate
+ Low

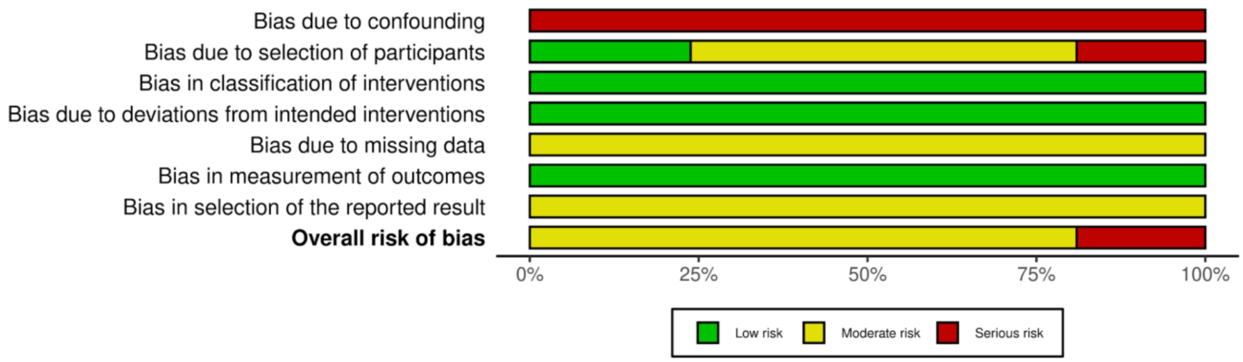

Figure S2.A: Risk of bias in the single arm clinical trials.

Study	Selection	Comparability	Outcome	Total	Quality
Popat et al. 2025	★★★		★★★	6	Moderate
Pasvolsky et al.2025	★★★		★★★	6	Moderate
Frenking et al. 2025	★★★		★★★	6	Moderate
Al hadidi et al. 2025	★★★		★★★	6	Moderate
Dhakal et al. 2024	★★★		★★★	6	Moderate
Gill et al. 2024	★★★		★★★	6	Moderate
Neupane et. al. 2025	★★★		★★★	6	Moderate
Perot et al. 2025	★★★		★★★	6	Moderate
Tan et al. 2025	★★★		★★★	6	Moderate
Perrot et al. 2024	★★★		★★★	6	Moderate
Reidhammer et al. 2024	★★★		★★★	6	Moderate
Mohan et al. 2023	★★★		★★★	6	Moderate
Dima et al. 2023	★★★		★★★	6	Moderate
Malard et al. 2024	★★★		★★★	6	Moderate
Portuguese et al. 2025	★★★		★★★	6	Moderate

Figure S2.B: Risk of bias in retrospective cohort studies.

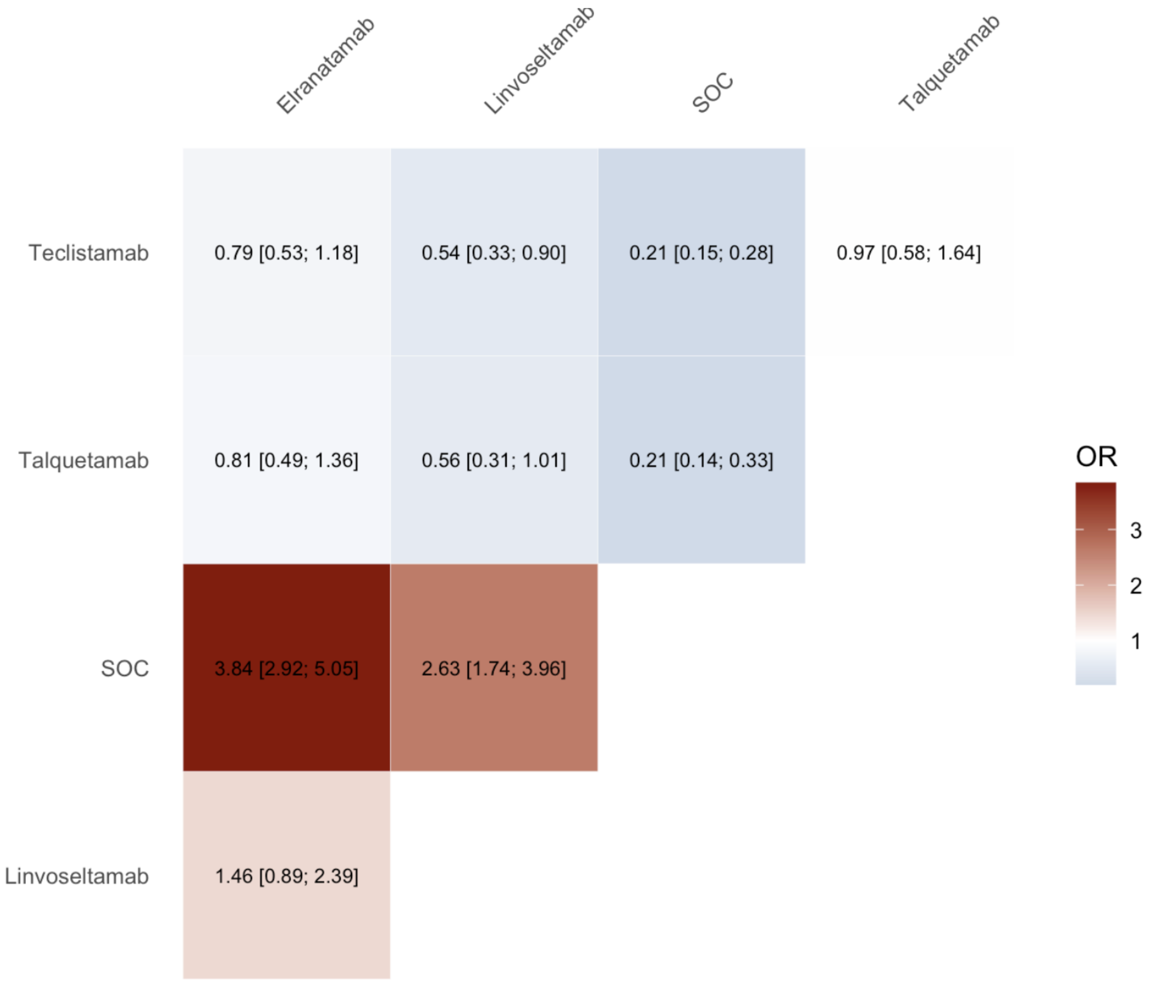

Figure S3: Network heat map comparing ORR of bispecific agents versus SOC in BCMA-naive group.

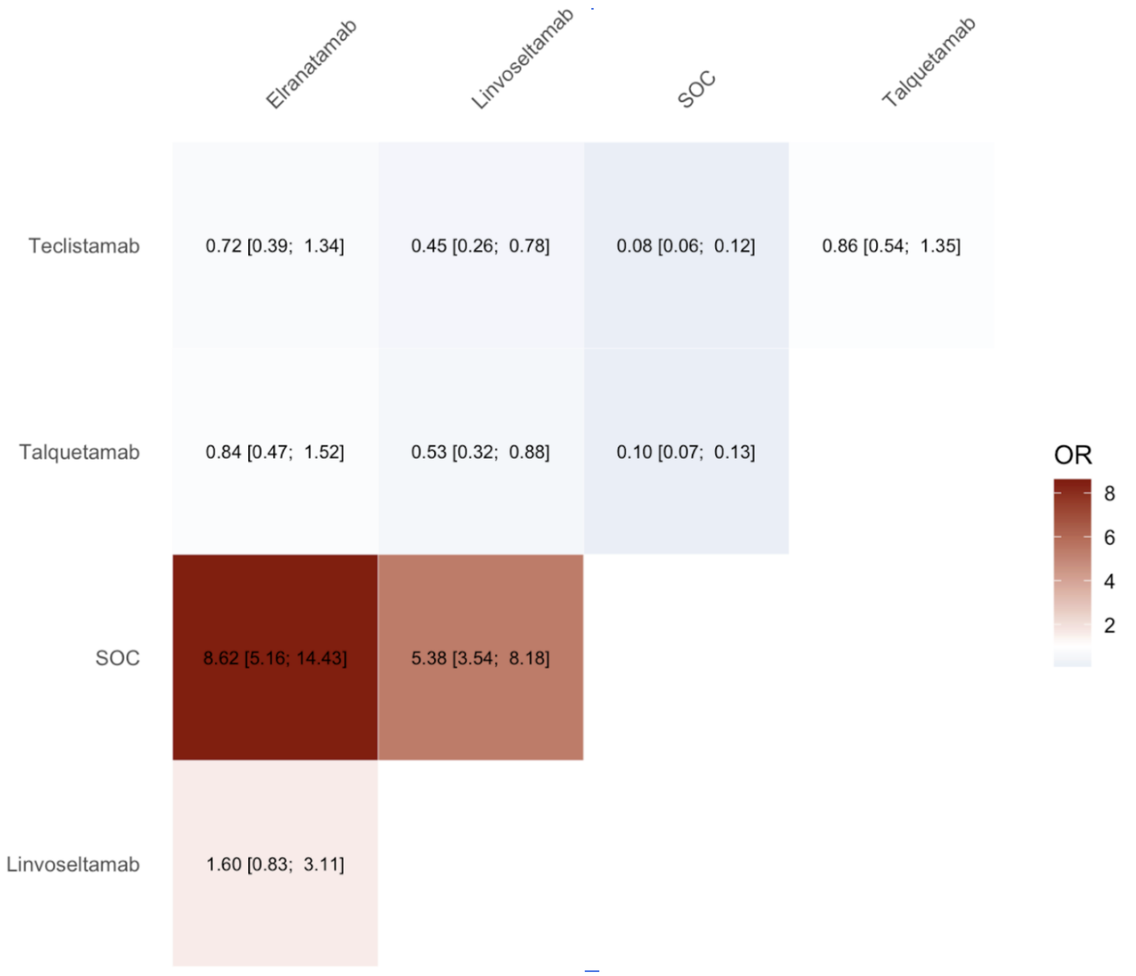

Figure S4: Network heat map of pairwise odds ratio for comparing ≥ VGPR comparing Bispecific agents and SOC regimens.

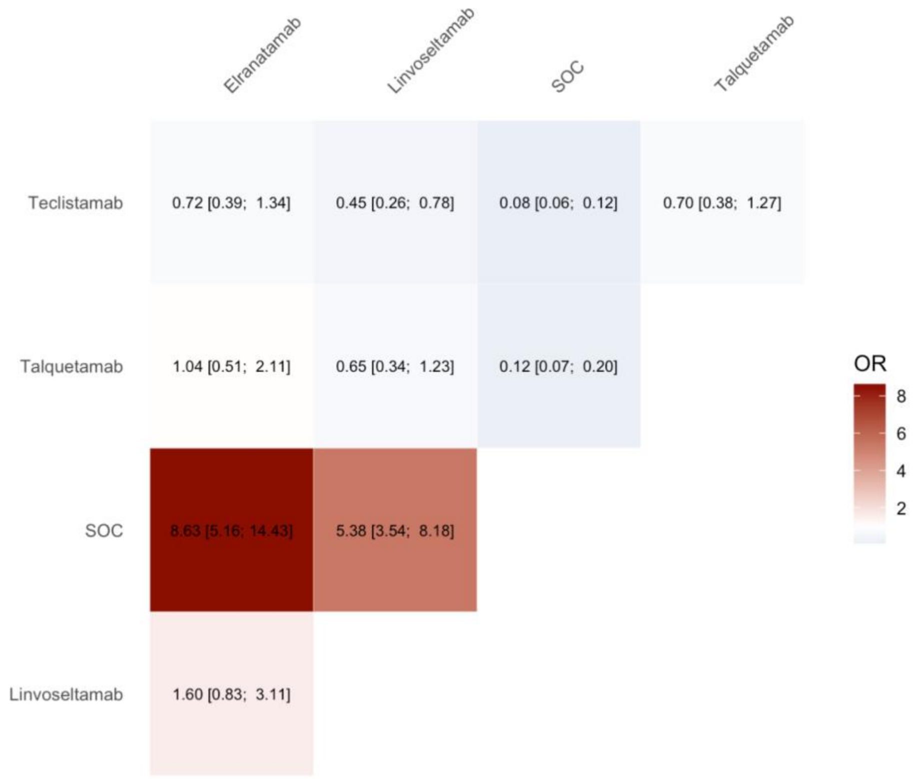

Figure S5: Network heat map comparing ≥ VGPR of bispecific agents versus SOC in BCMA-naive group.

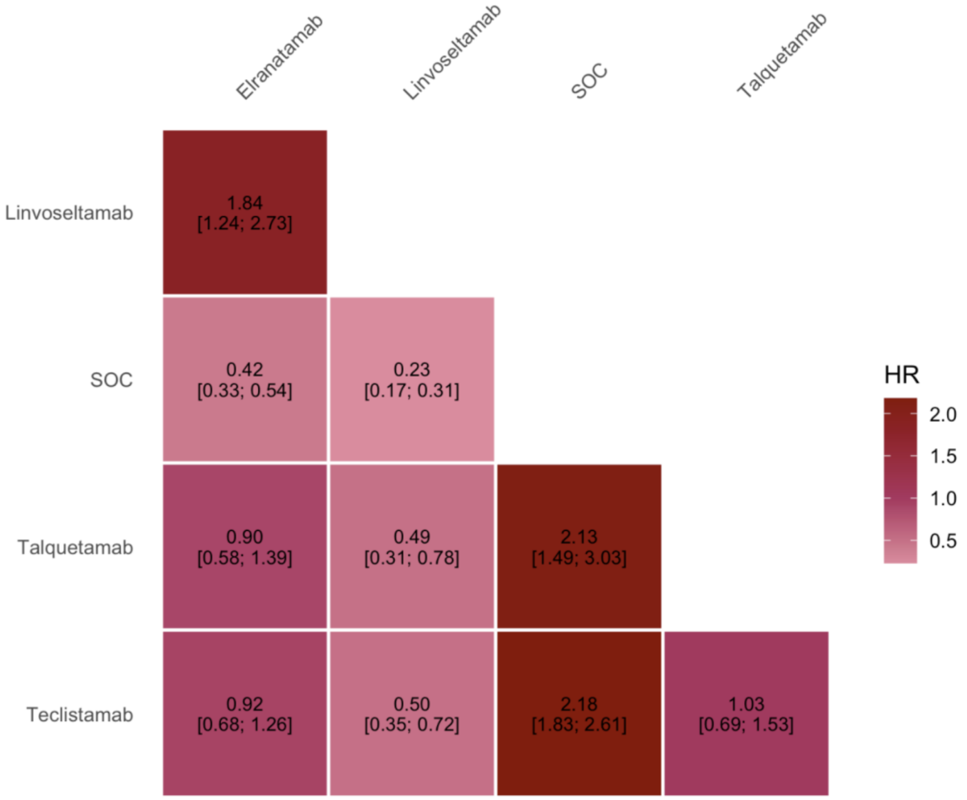

Figure S6: Network heat map comparing PFS of bispecific agents versus SOC in BCMA-naive group.

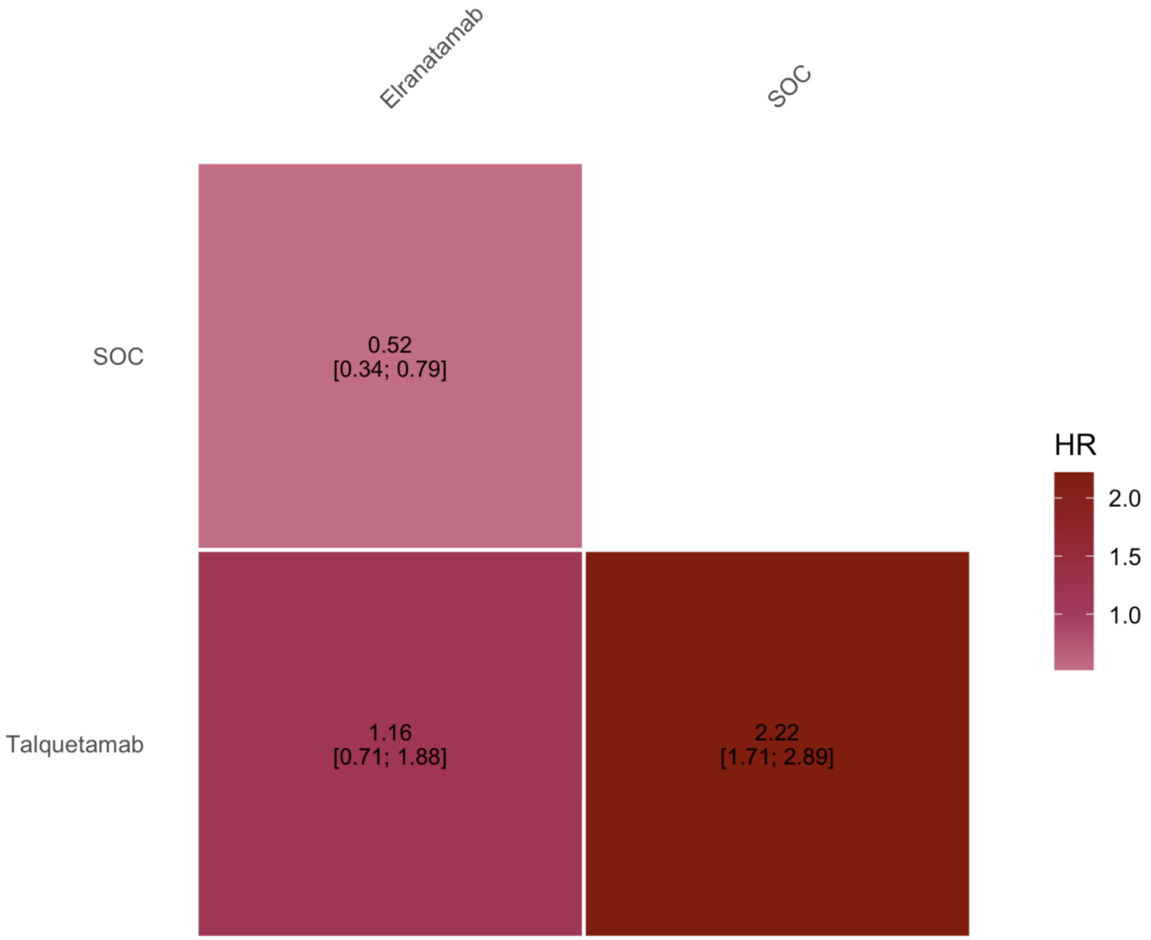

Figure S7: Network heat map comparing PFS of bispecific agents versus SOC in BCMA-exposed group.

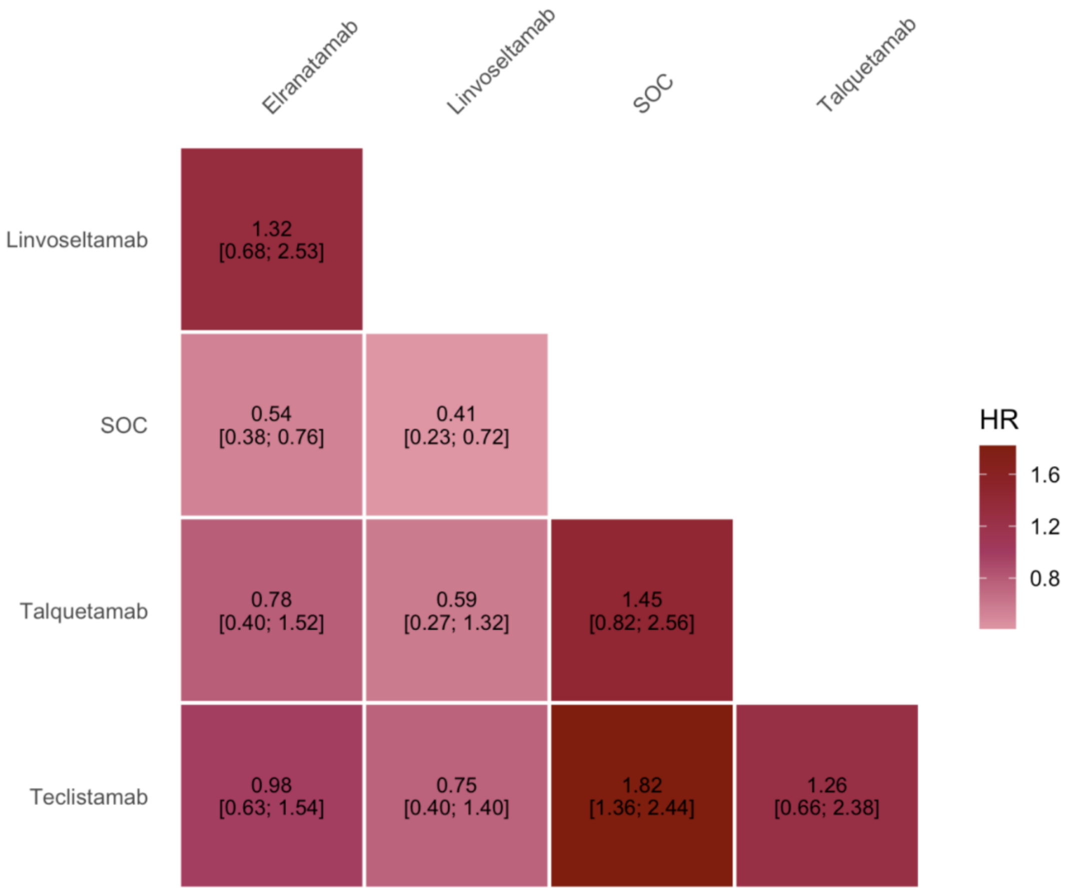

Figure S8: Network heat map comparing OS of bispecific agents versus SOC in BCMA-naive group.

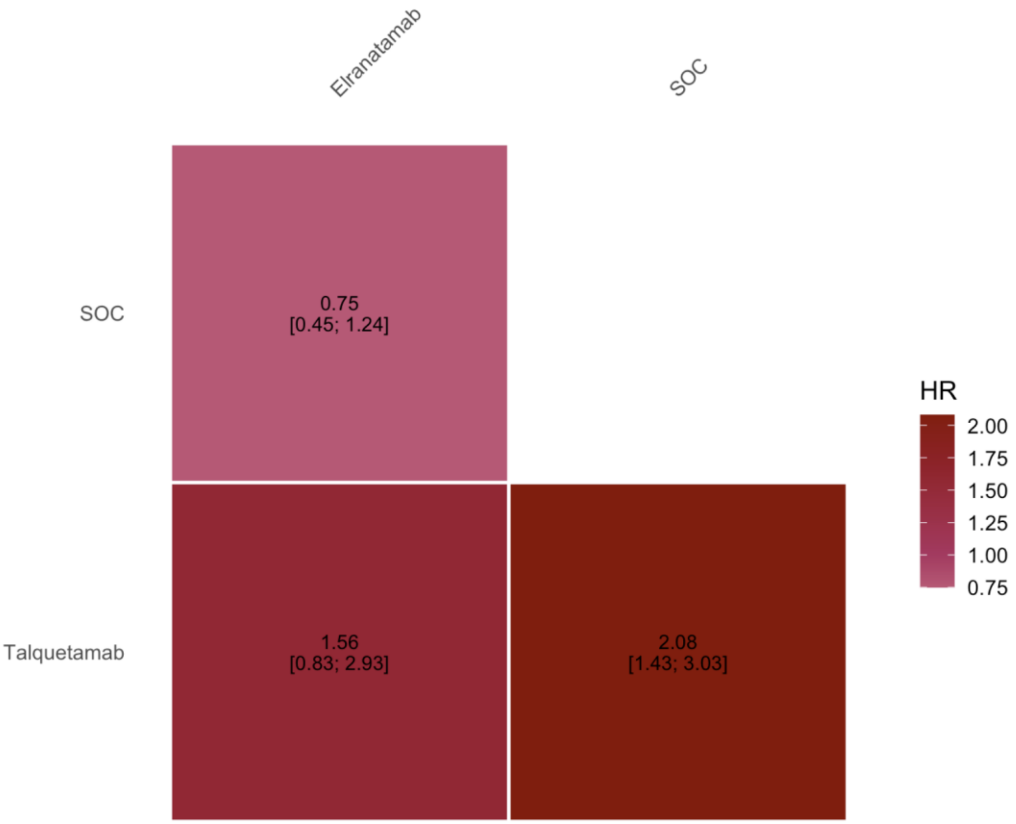

Figure S9: Network heat map comparing OS of bispecific agents versus SOC in BCMA-exposed group.

<u>Pooled single arm analysis of clinical trials and retrospective studies:</u>

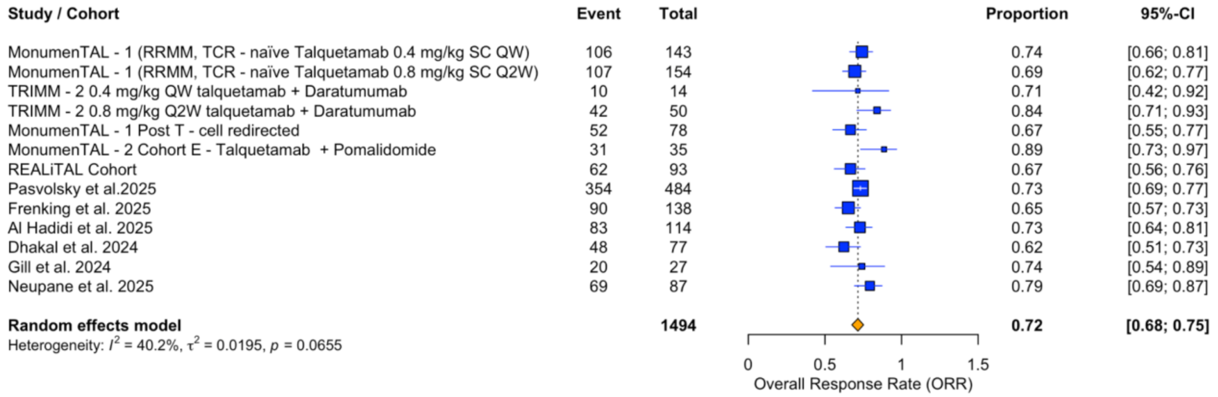

Figure S10: Objective Response Rate (ORR) - Talquetamab.

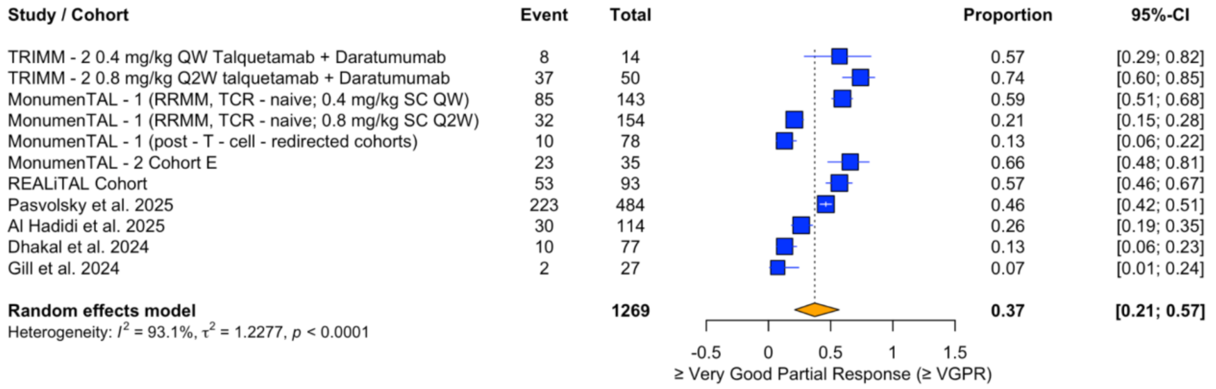

Fig S11: ≥ Very Good Partial Response (≥ VGPR) – Talquetamab.

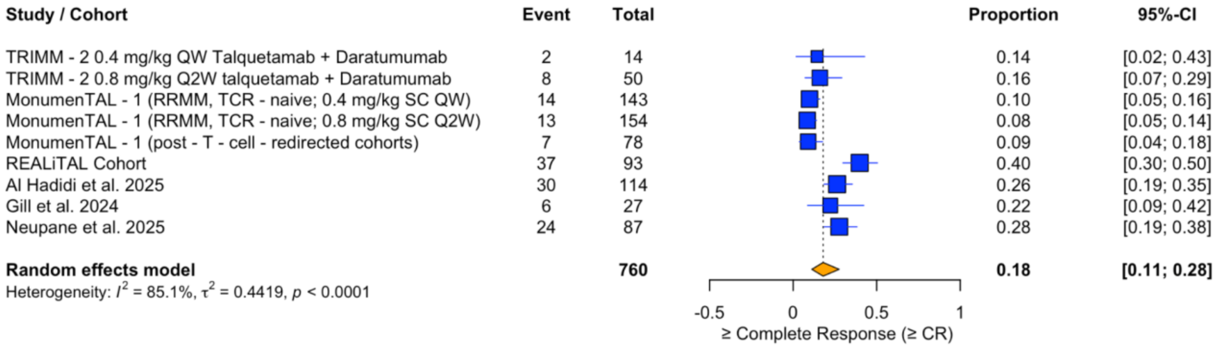

Fig S12: ≥ Complete Response (≥ CR) – Talquetamab.

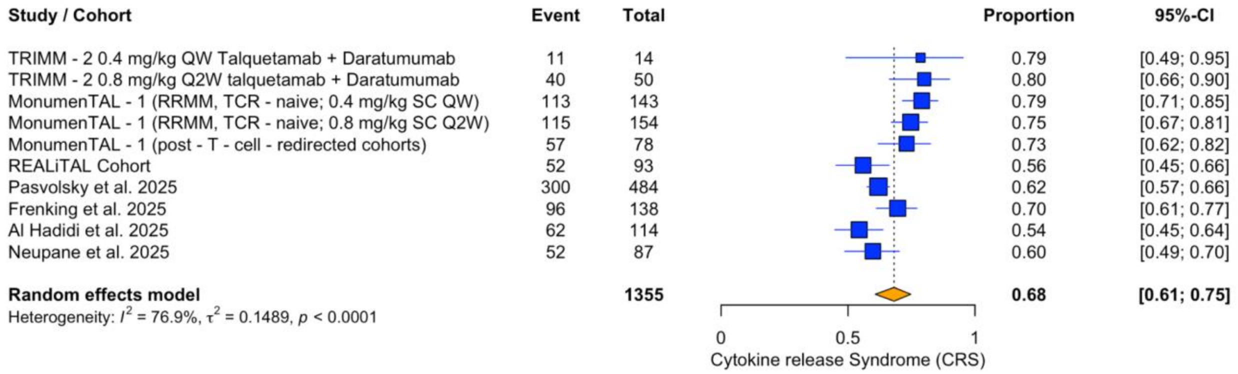

Figure S13: Cytokine release syndrome (CRS) - Talquetamab.

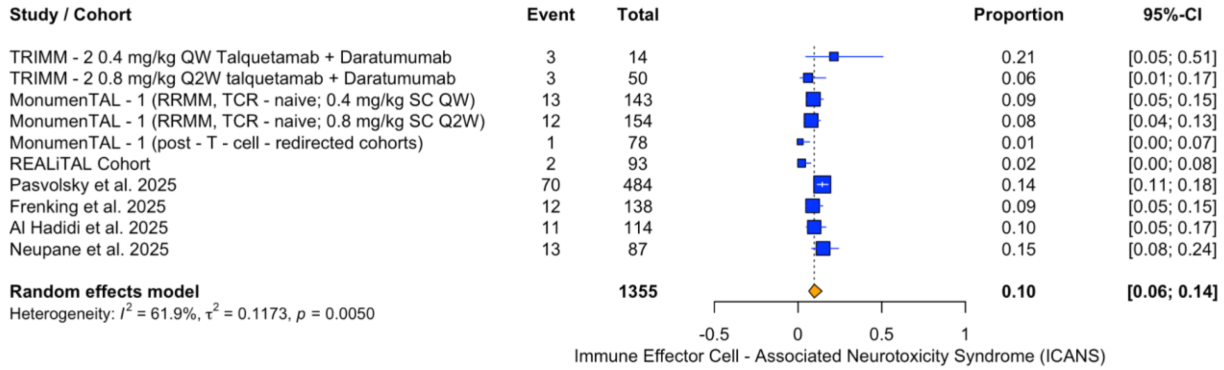

Fig S14: Immune Cell - Associated Neurotoxicity Syndrome (ICANS) - Talquetamab.

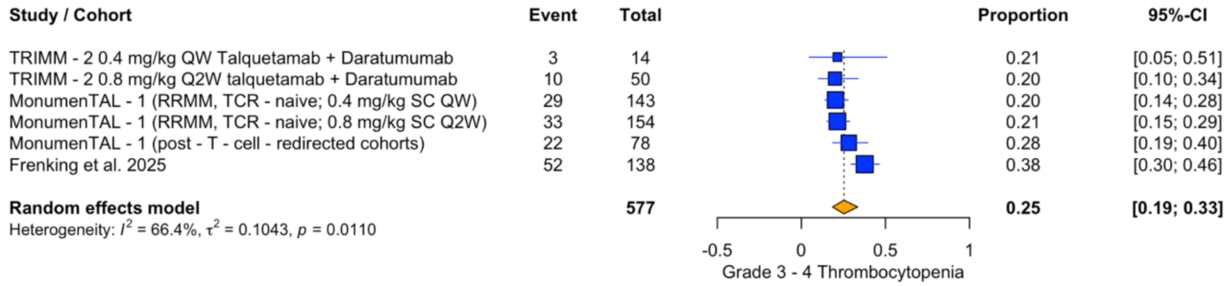

Fig S15: Grade 3 - 4 Thrombocytopenia - Talquetamab.

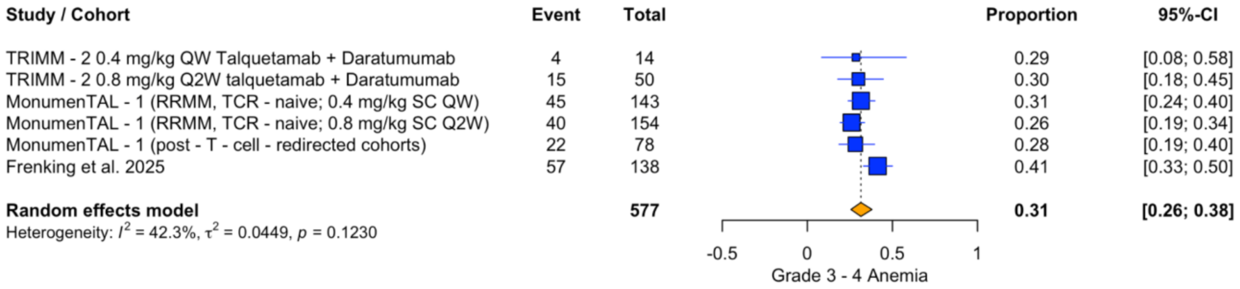

Figure S16: Grade 3 - 4 Anemia - Talquetamab.

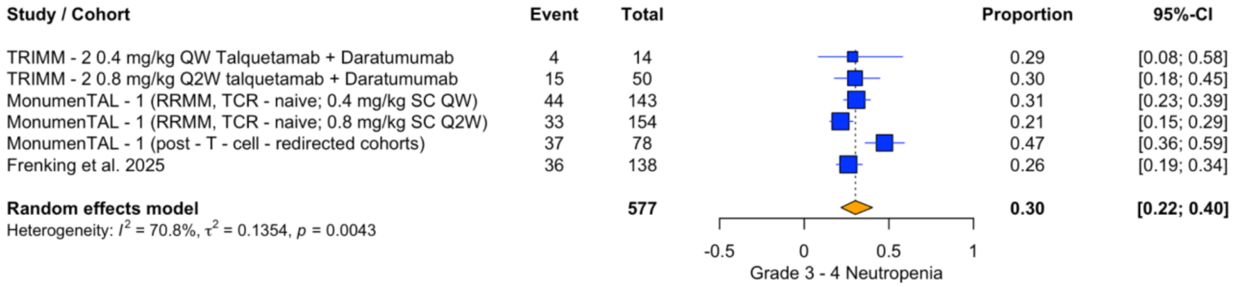

Figure S17: Grade 3 - 4 Neutropenia - Talquetamab.

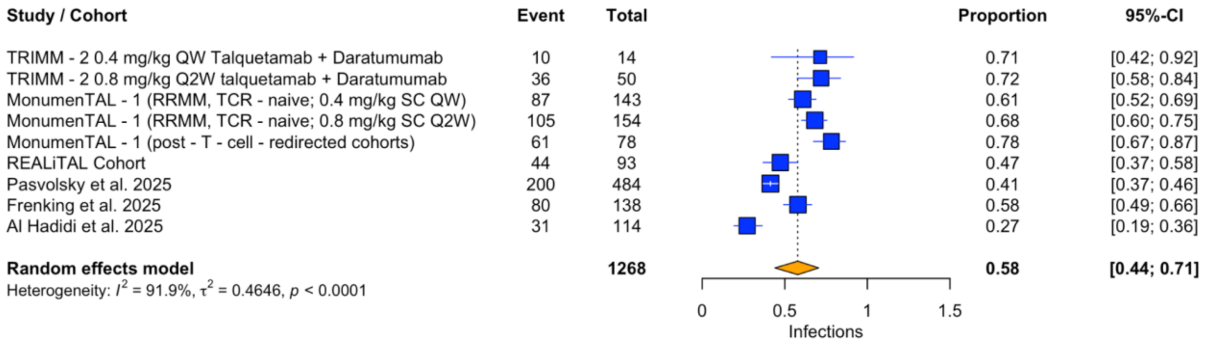

Figure S18: Infections - Talquetamab.

Teclistamab:

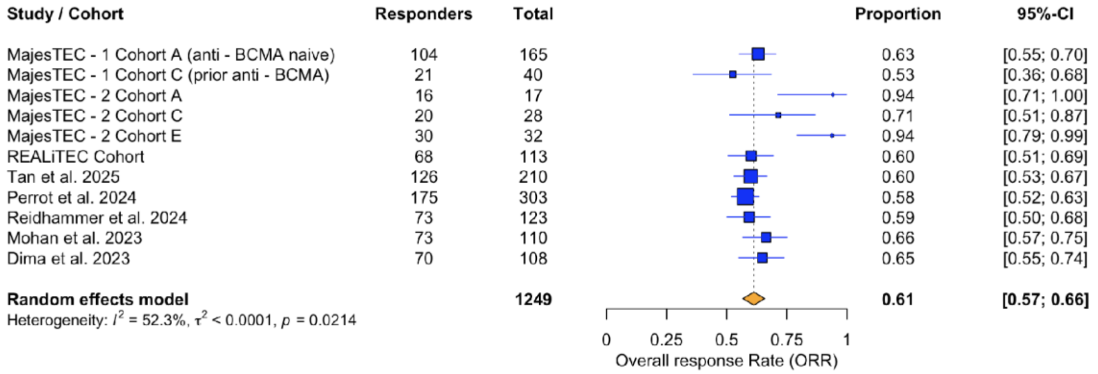

Figure S19: Objective Response Rate (ORR) - Teclistamab.

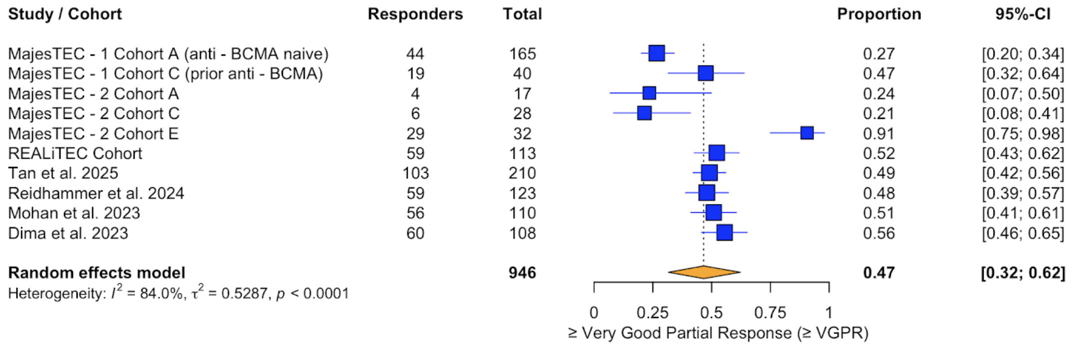

Figure S20: ≥ Very Good Partial Response (≥ VGPR) – Teclistamab.

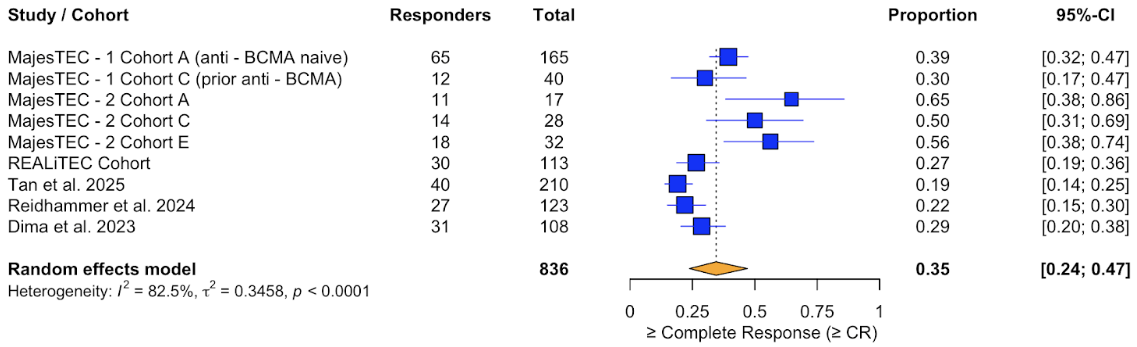

Figure S21: ≥ Complete Response (≥ CR) – Teclistamab.

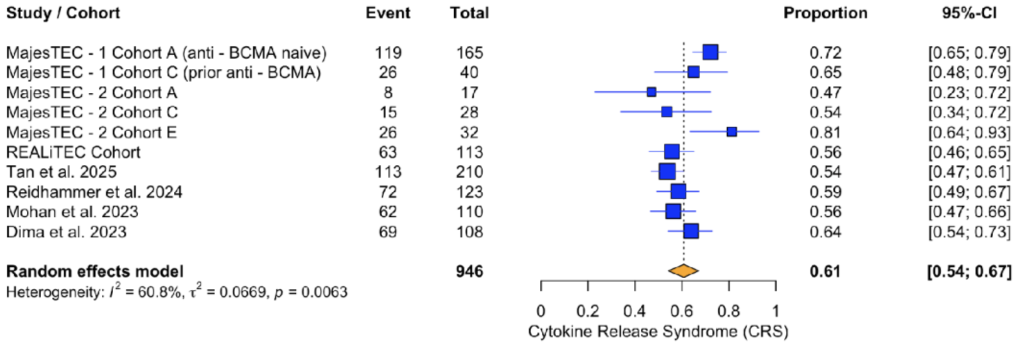

Figure S22: Cytokine release syndrome (CRS) - Teclistamab.

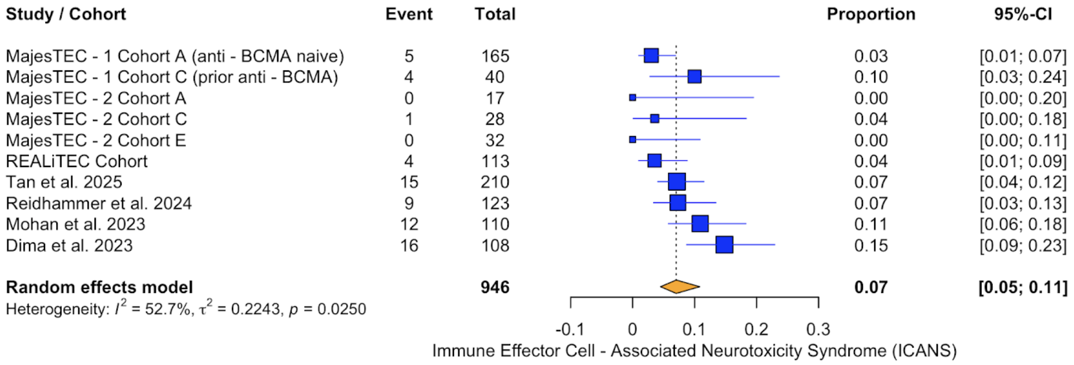

Figure S23: Immune Cell - Associated Neurotoxicity Syndrome (ICANS) - Teclistamab.

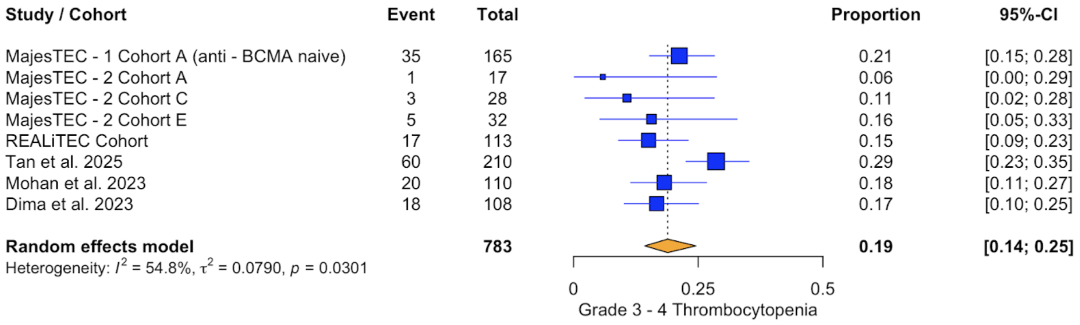

Figure S24: Grade 3 - 4 Thrombocytopenia - Teclistamab.

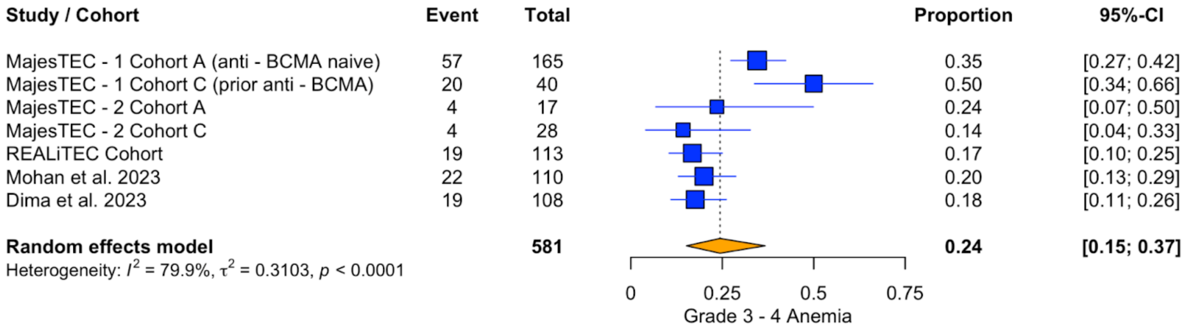

Figure S25: Grade 3 - 4 Anemia - Teclistamab.

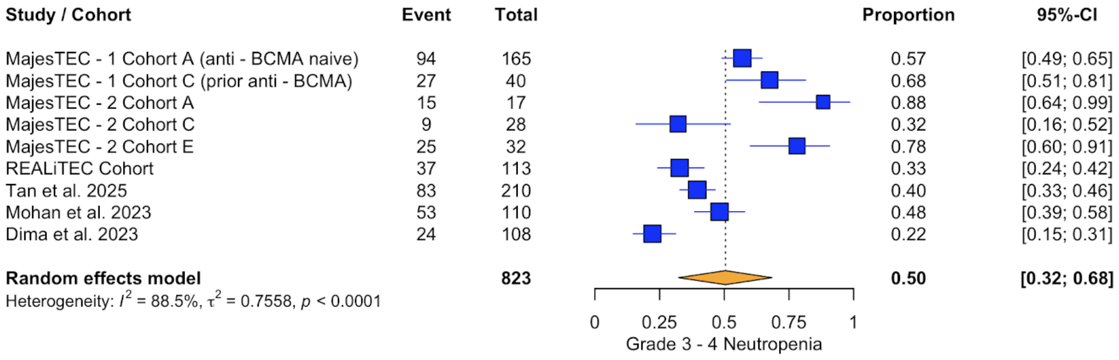

Figure S26: Grade 3 - 4 Neutropenia - Teclistamab.

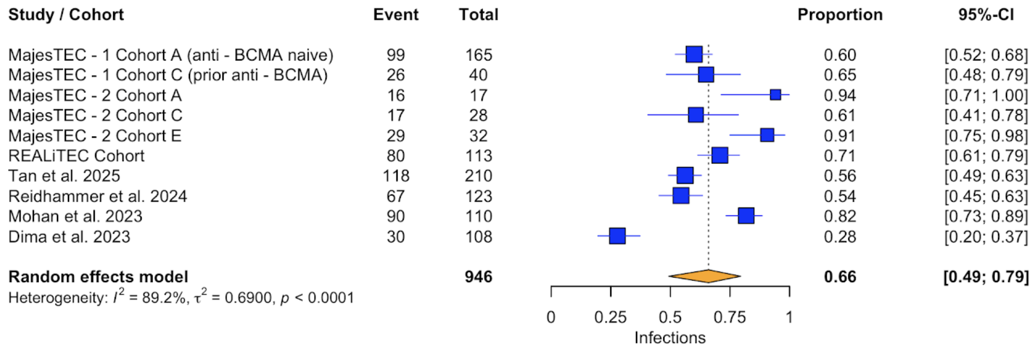

Figure S27: Infections - Teclistamab.

Cevostamab

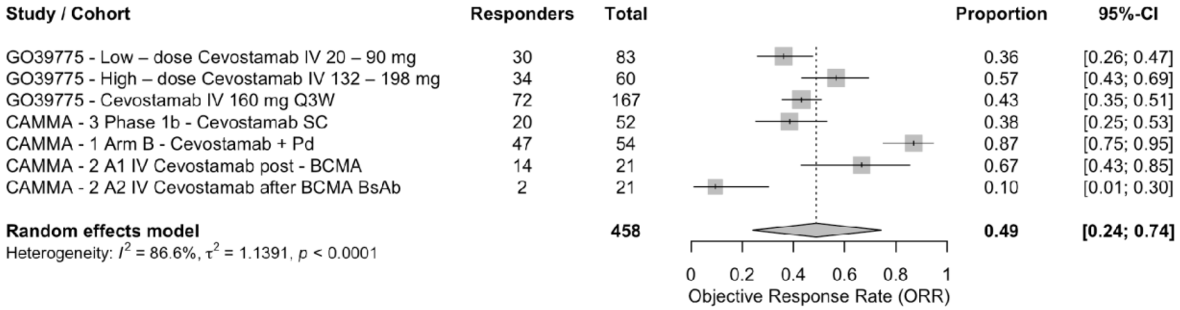

Figure S28: Overall response rate (ORR) - Cevostamab.

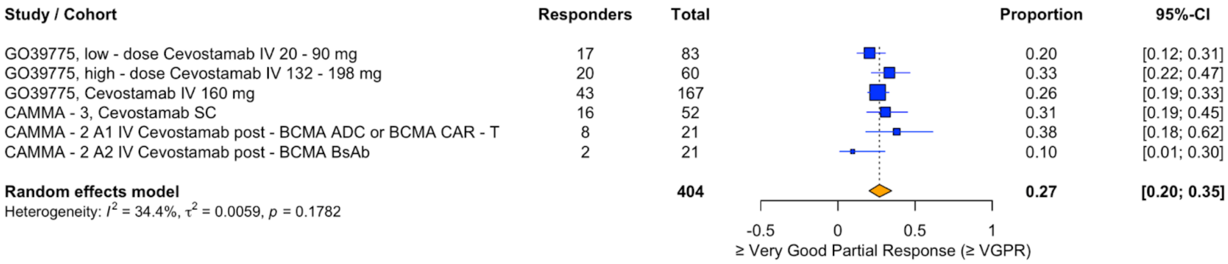

Figure S29: ≥ Very Good Partial Response (≥ VGPR) – Cevostamab.

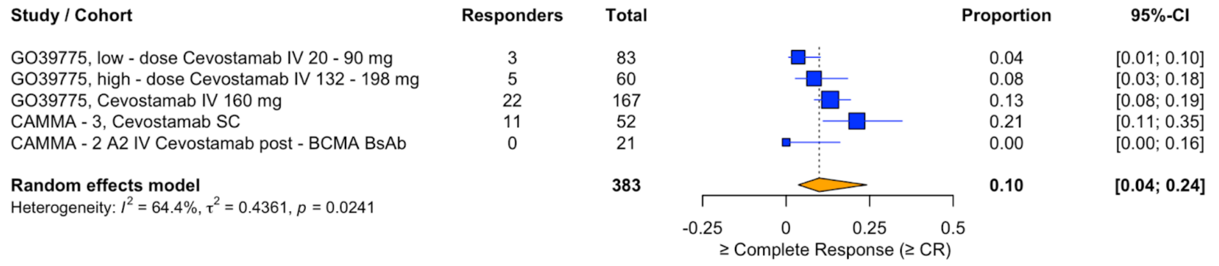

Figure S30: ≥ Complete Response (≥ CR) – Cevostamab.

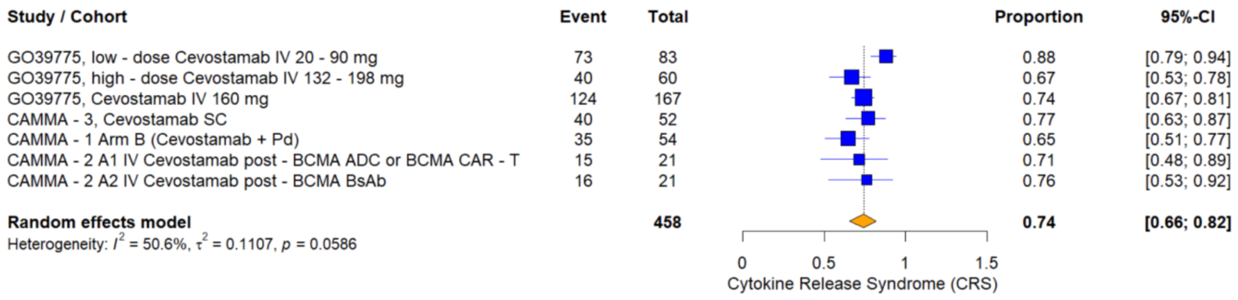

Figure S31: Cytokine Release Syndrome (CRS) - Cevostamab.

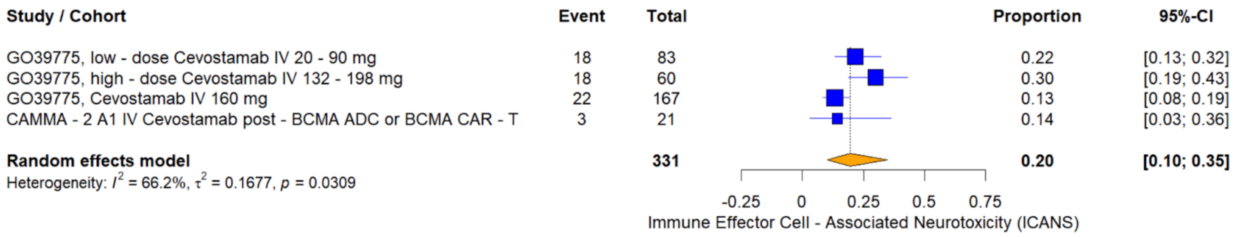

Figure S32: Immune Cell - Associated Neurotoxicity Syndrome (ICANS) - Cevostamab.

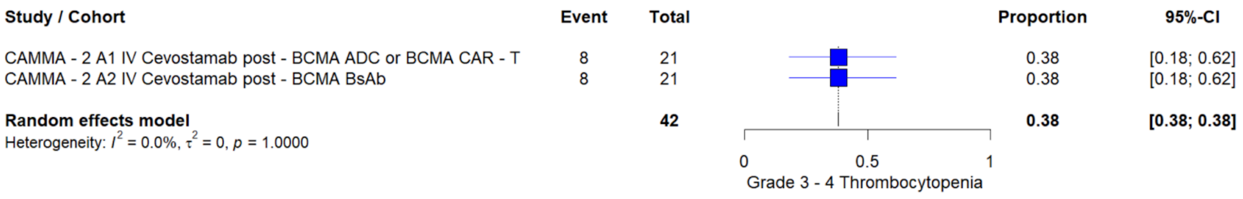

Figure S33: Grade 3 - 4 Thrombocytopenia - Cevostamab.

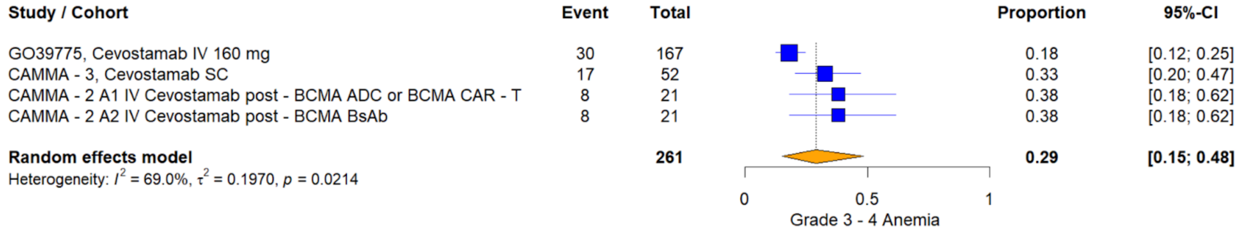

Figure S34: Grade 3 - 4 Anemia - Cevostamab.

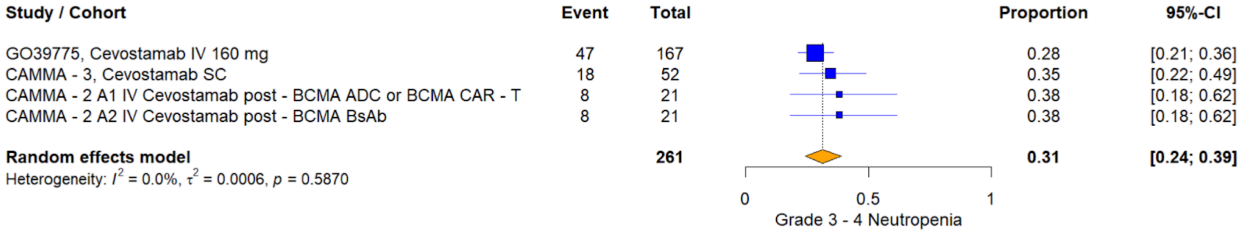

Figure S35: Grade 3 - 4 Neutropenia - Cevostamab.

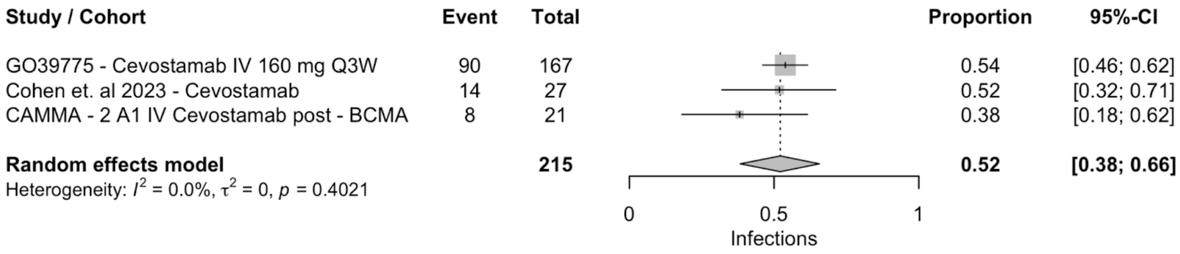

Figure S36: Infections - Cevostamab.

Elranatamab

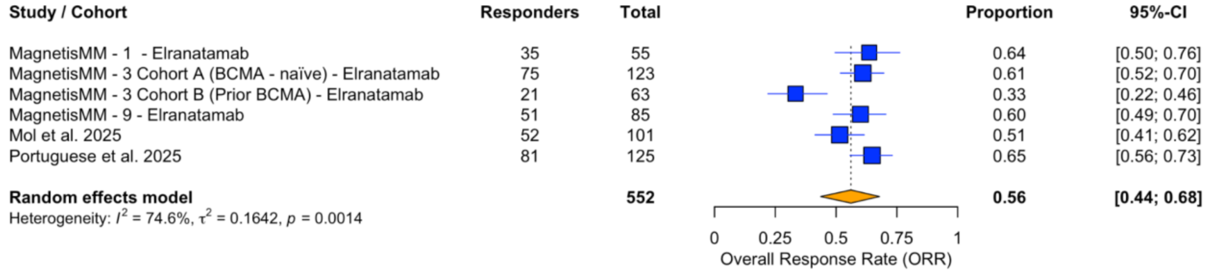

Figure S37: Overall Response Rate (ORR) - Elranatamab.

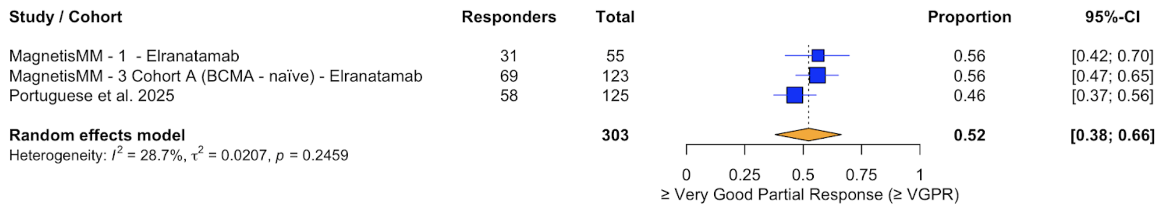

Figure S38: ≥ Very Good Partial Response (≥ VGPR) – Elranatamab.

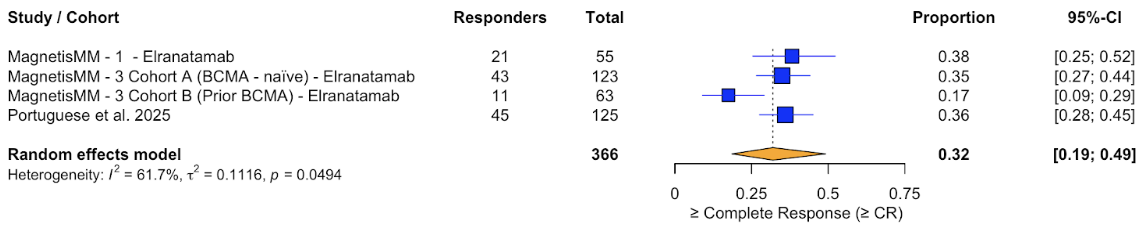

Figure S39: ≥ Complete Response (≥ CR) – Elranatamab.

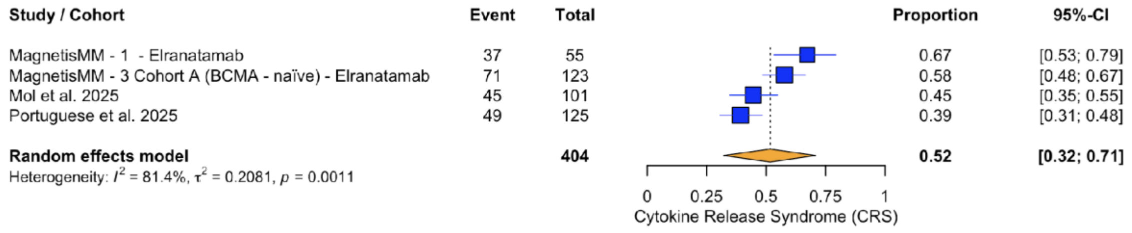

Figure S40: Cytokine Release Syndrome (CRS) - Elranatamab.

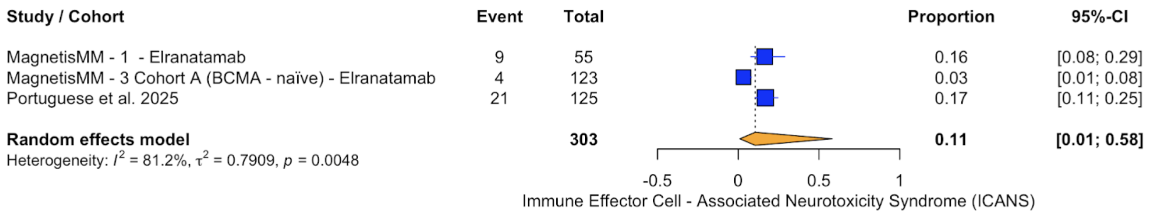

Figure S41: Immune Cell - Associated Neurotoxicity Syndrome (ICANS) - Elranatamab.

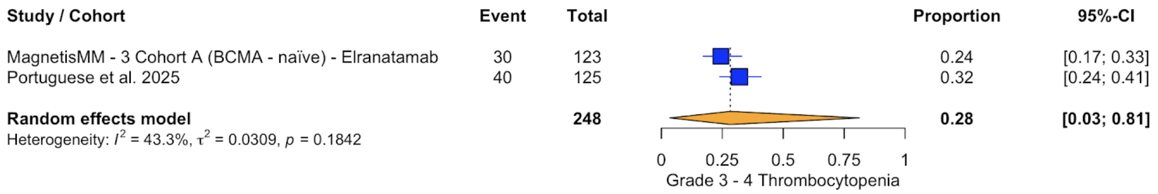

Figure S42: Grade 3 - 4 Thrombocytopenia - Elranatamab.

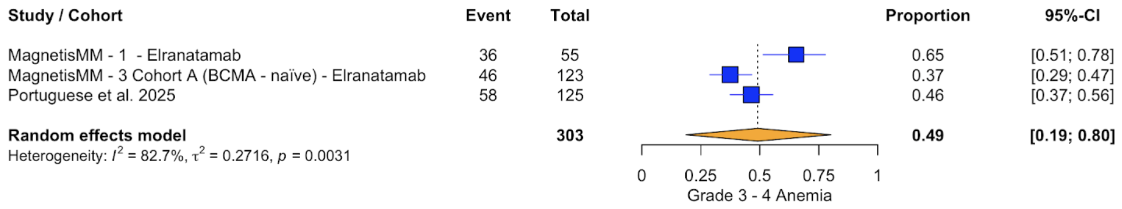

Figure S43: Grade 3 - 4 Anemia - Elranatamab.

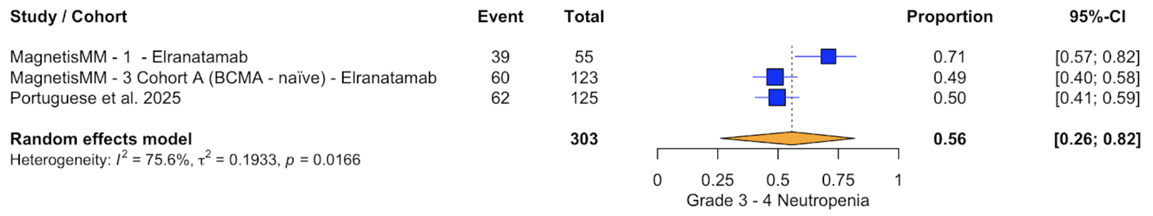

Figure S44: Grade 3 - 4 Neutropenia - Elranatamab.

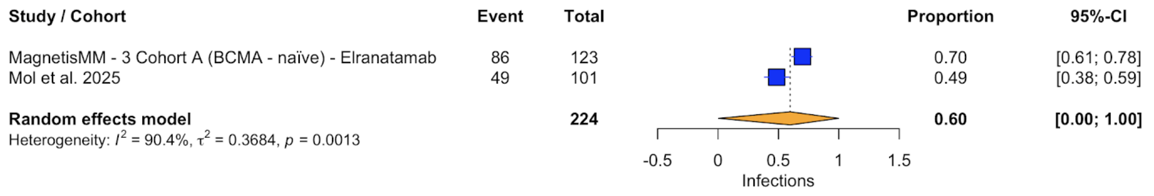

Figure S45: Infections - Elranatamab.

Linvoseltamab

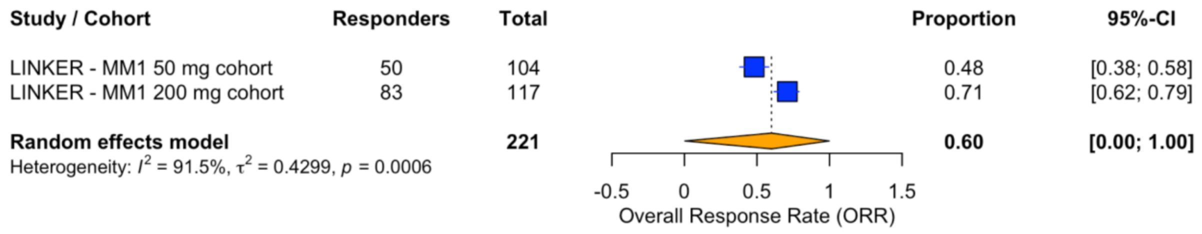

Fig S46: Overall Response Rate (ORR) - Linvoseltamab.

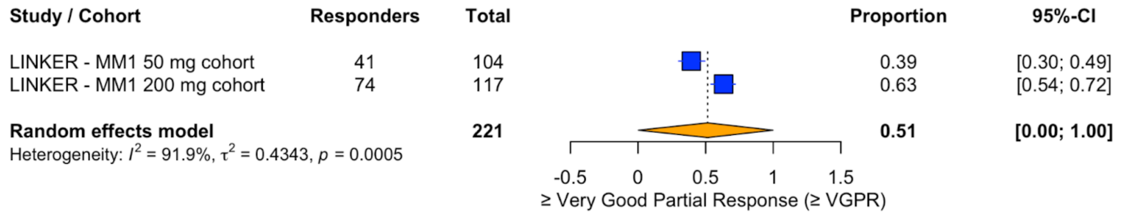

Figure S47: ≥ Very Good Partial Response (≥ VGPR) – Linvoseltamab.

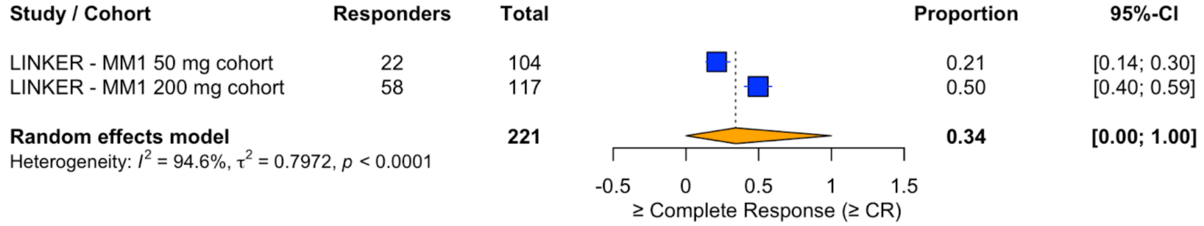

Figure S48: ≥ Complete Response (≥ CR) – Linvoseltamab.

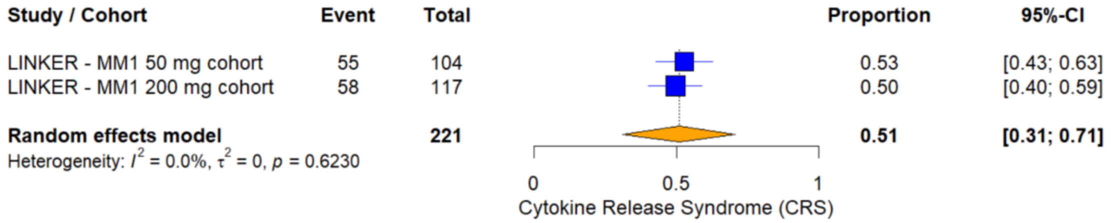

Figure S49: Cytokine Release Syndrome (CRS) - Linvoseltamab.

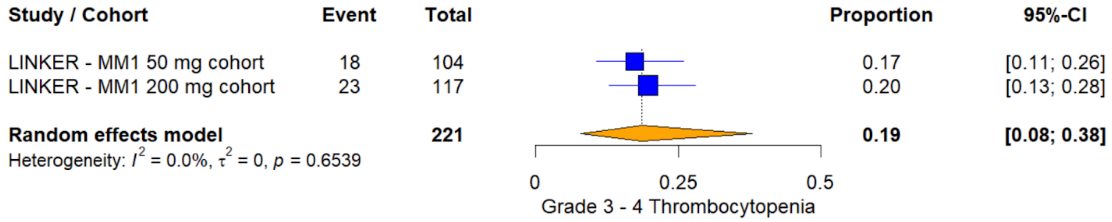

Figure S50: Grade 3 - 4 Thrombocytopenia - Linvoseltamab.

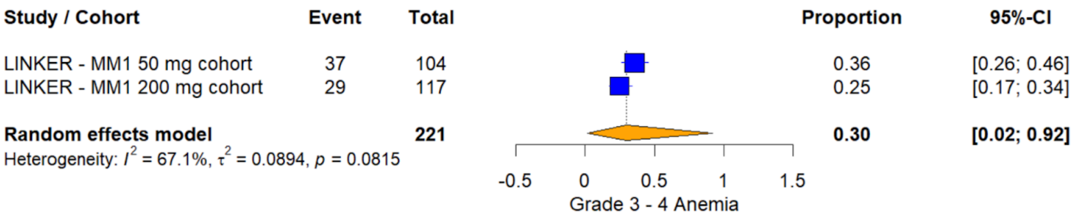

Figure S51: Grade 3 - 4 Anemia - Linvoseltamab.

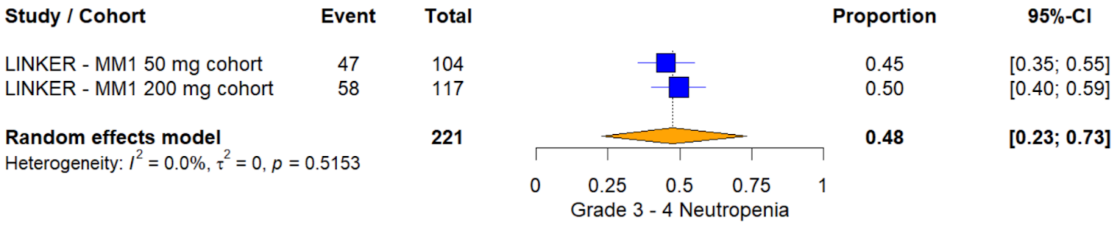

Figure S52: Grade 3 - 4 Neutropenia - Linvoseltamab.

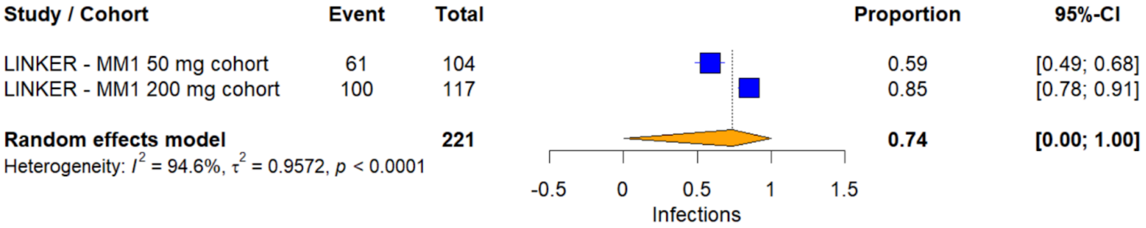

Figure S53: Infections - Linvoseltamab.

Sensitivity analysis of single arm studies on the basis of prior BCMA exposure:

Talquetamab:

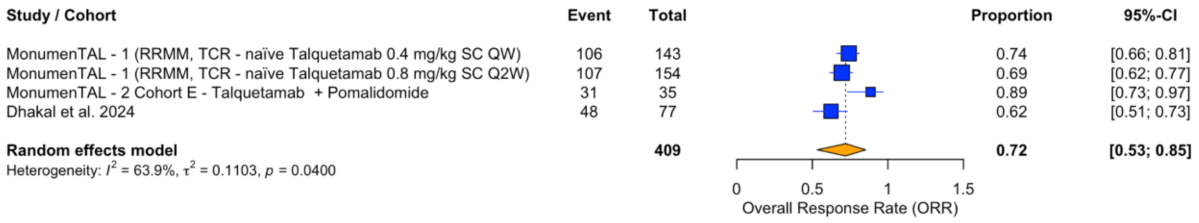

Figure S54: Forest plot depicting ORR in Talquetamab BCMA-naive group.

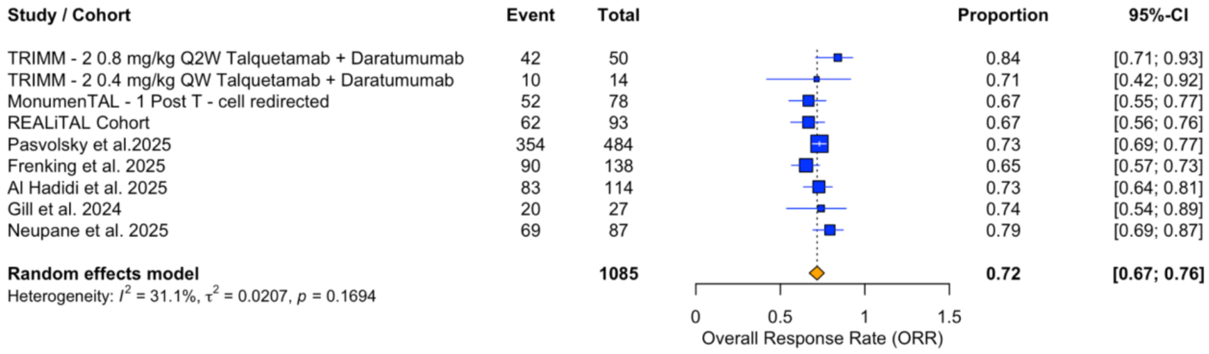

Figure S55: Forest plot depicting ORR in Talquetamab BCMA-exposed group.

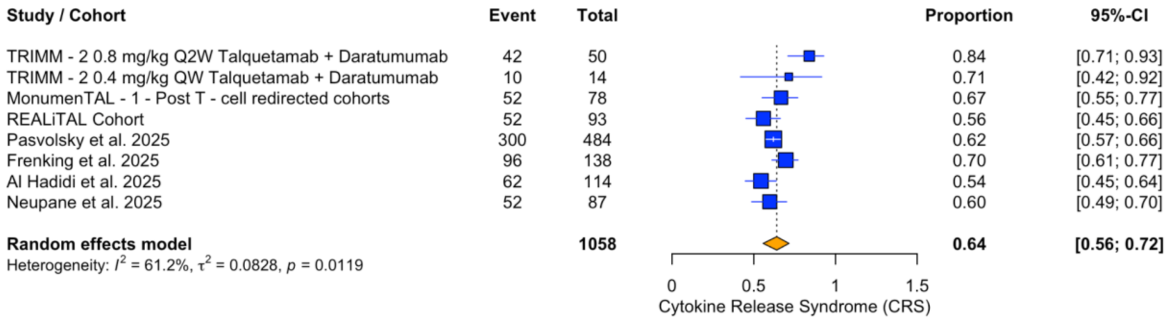

Figure S56: Forest plot depicting CRS in Talquetamab BCMA-exposed group.

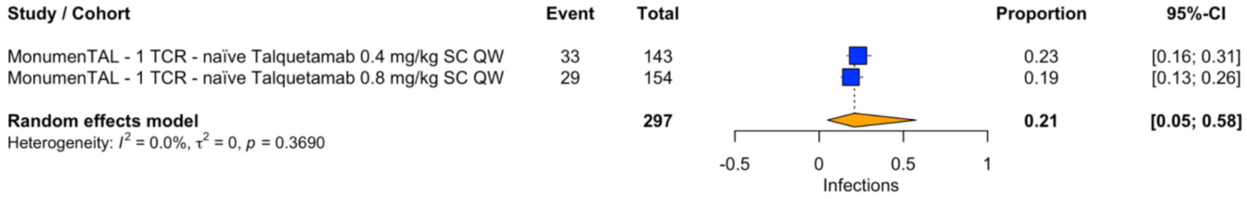

Figure S57: Forest plot depicting Infections in Talquetamab BCMA-naive group.

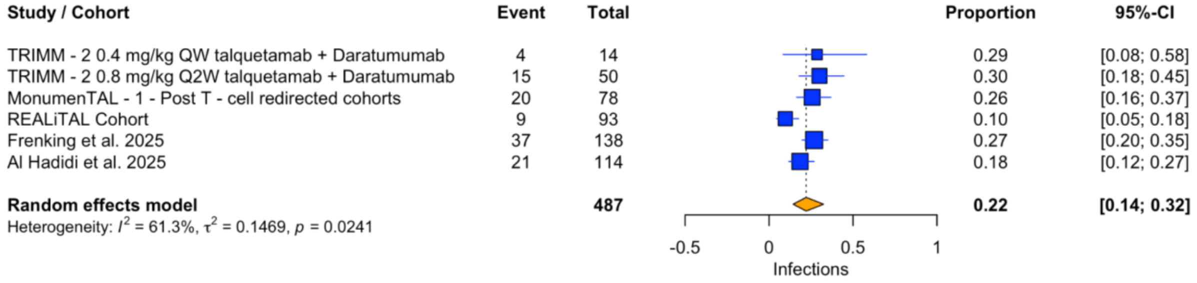

Figure S58: Forest plot depicting Infections in Talquetamab BCMA-exposed group.

Teclistamab:

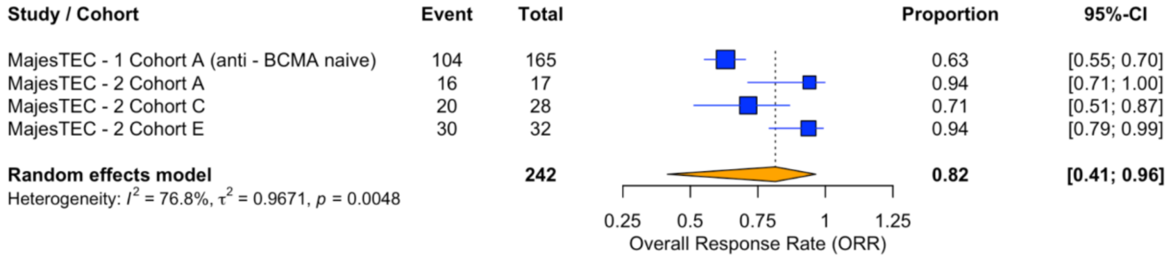

Figure S59: Forest plot depicting ORR in Teclistamab BCMA-naive group.

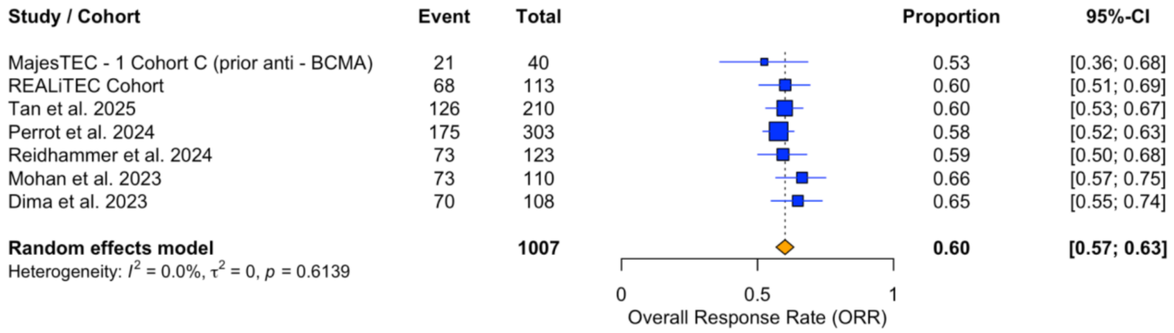

Figure S60: Forest plot depicting ORR in Teclistamab BCMA-exposed group.

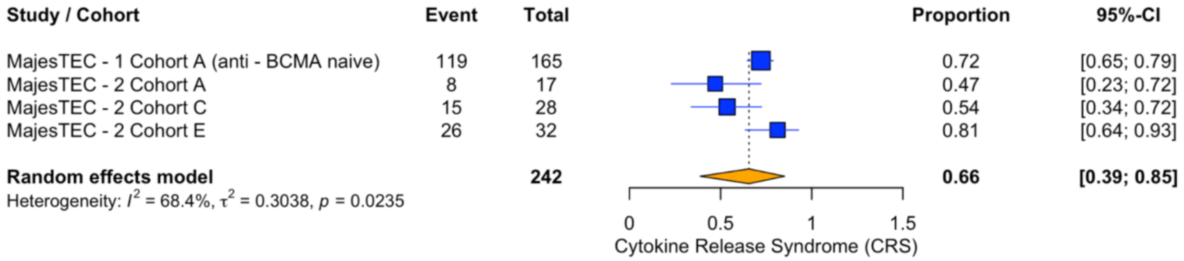

Figure S61: Forest plot depicting CRS in Teclistamab BCMA-naive group.

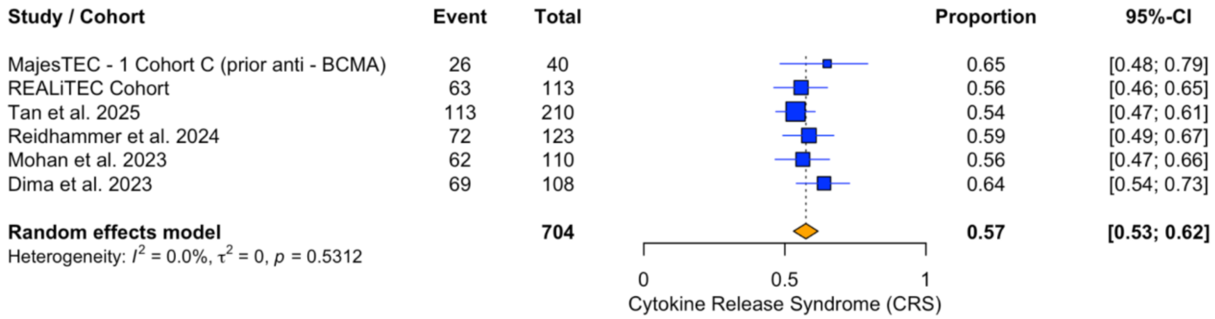

Figure S62: Forest plot depicting CRS in Teclistamab BCMA-exposed group.

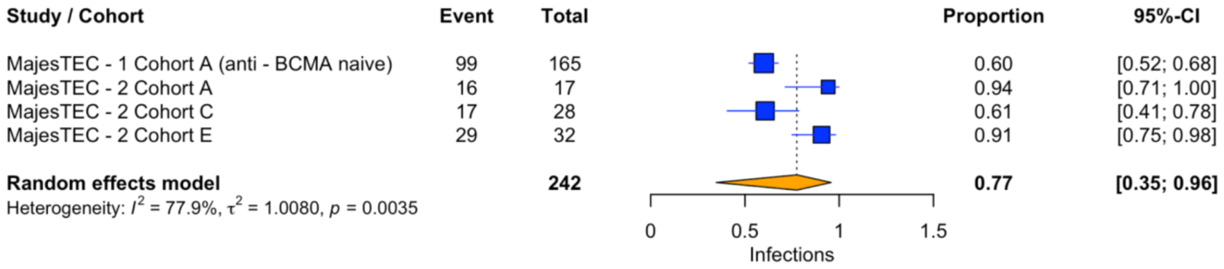

Figure S63: Forest plot depicting Infections in Teclistamab BCMA-naive group.

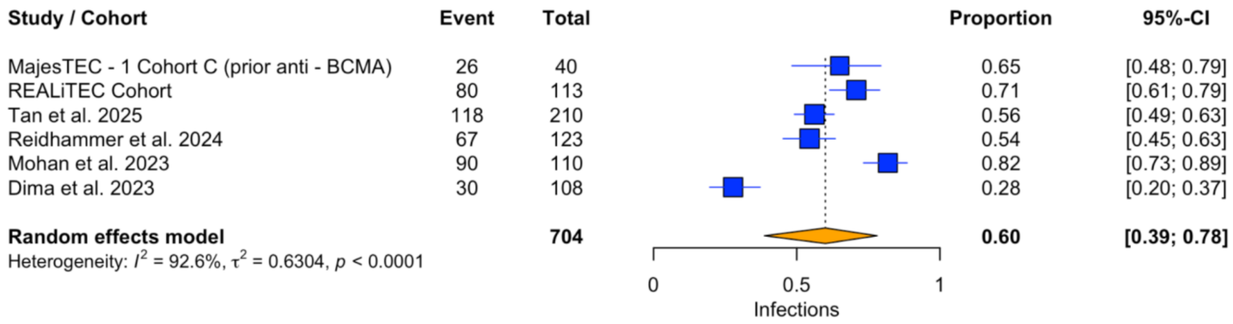

Figure S64: Forest plot depicting Infections in Teclistamab BCMA-exposed group.

Elranatamab:

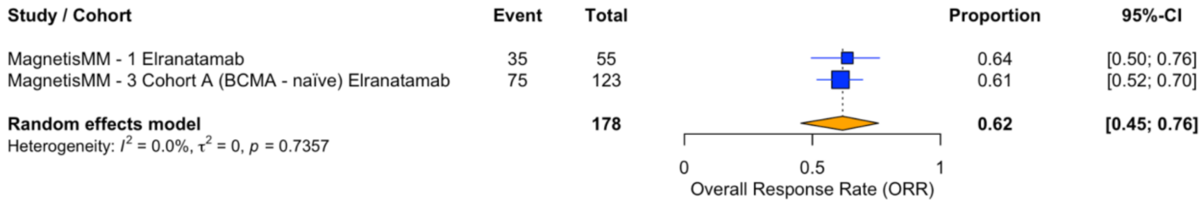

Figure S65: Forest plot depicting ORR in Elranatamab BCMA-naive group.

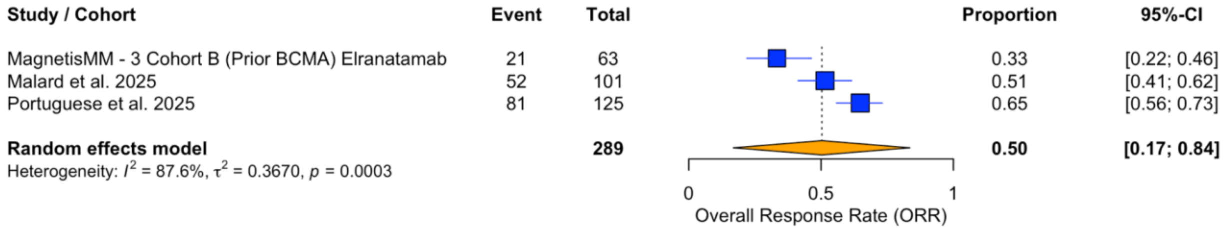

Figure S66: Forest plot depicting ORR in Elranatamab BCMA-exposed group.

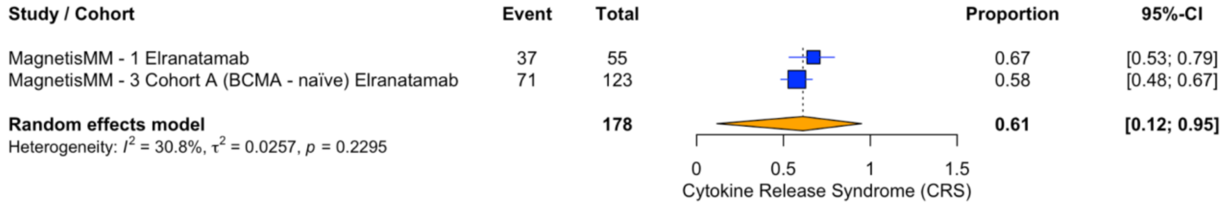

Figure S67: Forest plot depicting CRS in Elranatamab BCMA-naive group.

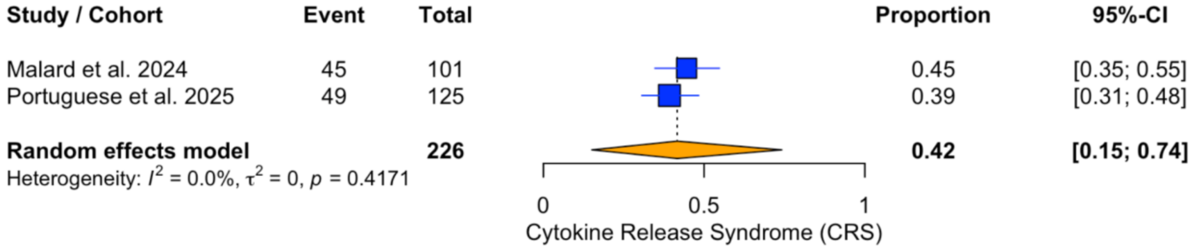

Figure S68: Forest plot depicting CRS in Elranatamab BCMA-exposed group.

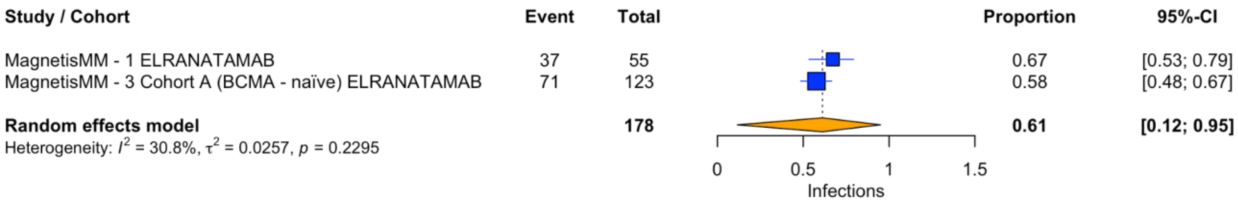

Figure S69: Forest plot depicting infections in Elranatamab BCMA-naive group.

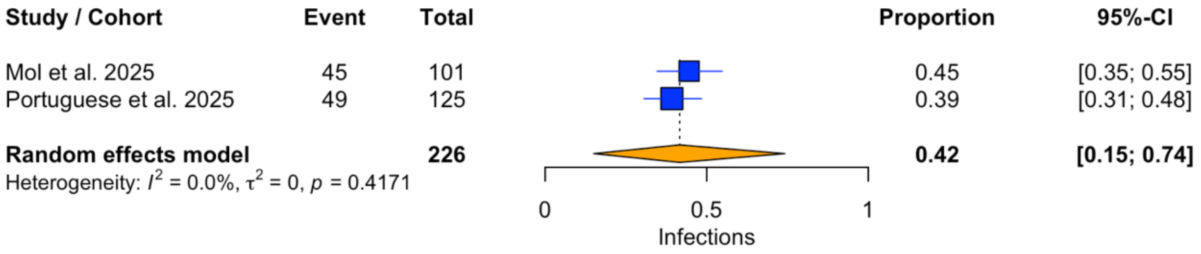

Figure S70: Forest plot depicting infections in Elranatamab BCMA-exposed group.

Cevostamab:

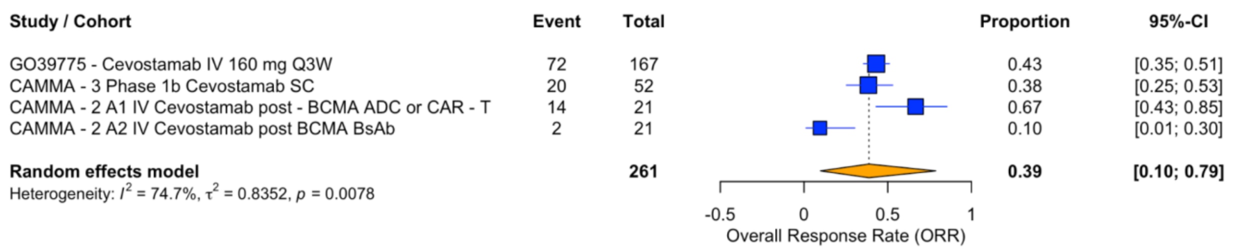

Figure S71: Forest plot depicting ORR in Cevostamab BCMA-naive group.

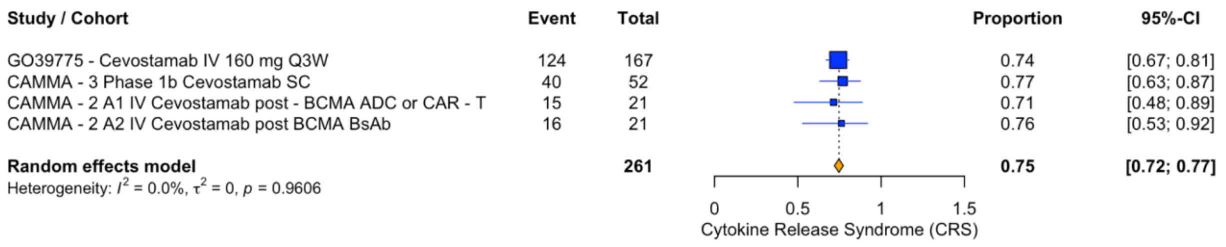

Figure S72: Forest plot depicting CRS in Cevostamab BCMA-naive group.

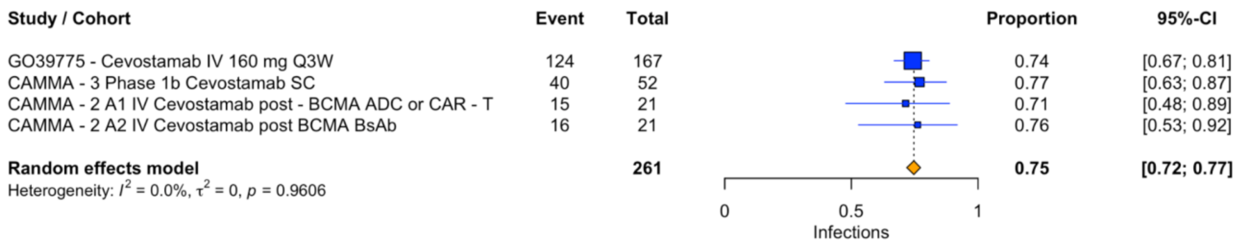

Figure S73: Forest plot depicting Infections in Cevostamab BCMA-naive group.
